# Liquid Biopsy: A Multi-Parametric Analysis of Mutation Status, Circulating Tumor Cells and Inflammatory Markers in *EGFR*-Mutated NSCLC

**DOI:** 10.3390/diagnostics12102360

**Published:** 2022-09-29

**Authors:** Martin P. Barr, Anne-Marie Baird, Sophia Halliday, Petra Martin, Emma H. Allott, James Phelan, Greg Korpanty, Linda Coate, Cathal O’Brien, Steven G. Gray, Jane S. Y. Sui, Brian Hayes, Sinead Cuffe, Stephen P. Finn

**Affiliations:** 1 Thoracic Oncology Research Group, Trinity St James’s Cancer Institute, St James’s Hospital, D08 W9RT Dublin, Ireland; 2 School of Medicine, Trinity Translational Medicine Institute, Trinity College Dublin, D08 W9RT Dublin, Ireland; 3 Patrick G. Johnston Centre for Cancer Research, Queen’s University Belfast, Belfast BT9 7AE, UK; 4 Department of Medical Oncology, Midlands Regional Hospital, R35 NY51 Tullamore, Ireland; 5 Department of Medical Oncology, University Hospital Limerick, V94 F858 Limerick, Ireland; 6 Cancer Molecular Diagnostics Laboratory, St James’s Hospital, D08 W9RT Dublin, Ireland; 7 Department of Histopathology, Cork University Hospital, T12 XF62 Cork, Ireland; 8 Department of Pathology, University College Cork, T12 DC4A Cork, Ireland; 9 Department of Medical Oncology, St James’s Hospital, D08 NHY1 Dublin, Ireland; 10 Department of Histopathology, St James’s Hospital, D08 RX0X Dublin, Ireland

**Keywords:** EGFR, liquid biopsy, T790M, biomarkers, CTCs, NGS, NSCLC

## Abstract

The liquid biopsy has the potential to improve patient care in the diagnostic and therapeutic setting in non-small cell lung cancer (NSCLC). Consented patients with epidermal growth factor receptor (*EGFR*) positive disease (*n* = 21) were stratified into two cohorts: those currently receiving *EGFR* tyrosine kinase inhibitor (TKI) therapy (*n* = 9) and newly diagnosed *EGFR* TKI treatment-naïve patients (*n* = 12). Plasma genotyping of cell-free DNA was carried out using the FDA-approved cobas^®^ *EGFR* mutation test v2 and compared to next generation sequencing (NGS) cfDNA panels. Circulating tumor cell (CTC) numbers were correlated with treatment response and *EGFR* exon 20 p.T790M. The prognostic significance of the neutrophil to lymphocyte ratio (NLR) and lactate dehydrogenase (LDH) was also investigated. Patients in cohort 1 with an *EGFR* exon 20 p.T790M mutation progressed more rapidly than those with an *EGFR* sensitizing mutation, while patients in cohort 2 had a significantly longer progression-free survival (*p* = 0.04). *EGFR* exon 20 p.T790M was detected by liquid biopsy prior to disease progression indicated by computed tomography (CT) imaging. The cobas^®^ *EGFR* mutation test detected a significantly greater number of exon 20 p.T790M mutations (*p* = 0.05). High NLR and derived neutrophil to lymphocyte ratio (dNLR) were associated with shorter time to progression and worse survival outcomes (*p* < 0.05). High LDH levels were significantly associated with shorter time to disease progression (*p* = 0.03). These data support the use of liquid biopsy for monitoring EGFR mutations and inflammatory markers as prognostic indicators in NSCLC.

## 1. Introduction

An improved understanding and identification of molecular pathways that drive tumor growth in non-small cell lung cancer (NSCLC) has led to the development of specific tyrosine kinase inhibitors (TKIs) in advanced stage disease. Mutations in the epidermal growth factor receptor (*EGFR*) tyrosine kinase domain occur more frequently in never smokers, the incidence of which is reported to be approximately 15% of adenocarcinoma cases and up to 62% in Asian populations [1,2]. In 2004, activating *EGFR* mutations were first identified in NSCLC and were characterized as oncogenic mutations that were shown to confer a more favourable prognosis and predict a greater sensitivity to *EGFR* TKIs [3,4,5]. In advanced NSCLC tumors of adenocarcinoma histology, current guidelines from the College of American Pathologists (CAP), the International Association for the Study of Lung Cancer (IASLC) and the Association of Molecular Pathologists (AMP) recommend *EGFR* analysis regardless of clinical characteristics [6], where these should not be used to either select or exclude patients for *EGFR* mutation testing, including ALK or ROS1 rearrangements.

*EGFR*, also known as ErbB1 or HER1, is a transmembrane receptor involved in the regulation of cell proliferation, survival, differentiation and other key cellular processes through the activation of multiple downstream signalling cascades, including the PI3K/AKT, RAS/RAF/MAPK and STAT pathways [7]. For the most part, *EGFR* gene mutations are located in the region encoding for the adenosine triphosphate (ATP) binding pocket of the kinase domain between exons 18–21. These result in the constitutive activation of the receptor tyrosine kinase domain, inducing downstream pro-survival signalling pathways. The most common *EGFR* mutations include in-frame exon 19 deletions and exon 21 point mutations, which result in the substitution of arginine for leucine at position 858 (L858R) and account for approximately 85% of *EGFR* mutations [8]. While additional *EGFR* sensitizing mutations such as G719X, L861Q and S768I have also been described [9,10,11], these mutations are relatively rare in NSCLC. For many years, first (e.g., gefitinib and erlotinib) or second-generation (e.g., afatinib) *EGFR* TKIs have been used successfully as first-line therapy in advanced *EGFR*-mutated NSCLC [12]. Nevertheless, acquired resistance inevitably develops in 50–60% of patients. This is due to the emergence of the secondary *EGFR* Thr790Met mutation in exon 20 (*EGFR* exon 20 p.T790M) of the *EGFR* gene [13] where osimertinib is indicated for use in this setting.

The use of targeted therapies such as *EGFR* TKIs has greatly advanced the personalized treatment of NSCLC patients. Traditionally, tissue genotyping has been used for identifying such genomic alterations. This methodology is however limited by a number of factors such as insufficient tumor tissue, tumor heterogeneity, inaccessibility of the tumor and risks associated with serial tumor biopsies, particularly in patients with underlying co-morbidities. Studies have shown that up to 20% of NSCLC tissue biopsies are inadequate for molecular testing due to inadequate DNA or insufficient tissue [14,15]. Plasma *EGFR* genotyping using circulating tumor DNA (ctDNA) is an emerging technology that has shown promise due to its accessibility, minimal invasiveness and potential clinical utility. To date, several platforms have been developed and clinically validated to examine their use in detecting and monitoring genomic alterations during the course of disease using liquid biopsy. These include amplification refractory mutation system PCR (ARMS-PCR), digital droplet PCR (ddPCR), BEAMing PCR and next-generation sequencing (NGS) [16,17,18,19,20]. Next generation sequencing (NGS) panels such as the AVENIO ctDNA Targeted Kit and Oncomine™ Lung cfDNA assays have largely been used for research use only. In 2016, the FDA approved the semi-quantitative PCR-based Roche cobas^®^ *EGFR* mutation test v2 as a companion diagnostic test, using ctDNA, for the detection of *EGFR*-sensitizing mutations in NSCLC. This approval was based on the ENSURE study, a multicentre, open-label, randomized Phase III study, evaluating the efficacy and safety of Tarceva versus gemcitabine plus cisplatin as first-line treatment for stage IIIB/IV NSCLC. Using paired plasma and tissue samples from 517 patients, the cobas^®^ test demonstrated moderate sensitivity and high specificity of 76.7% and 98.2%, respectively, for the detection of *EGFR*-sensitizing mutations [21]. In early phase, multicentre trials (AURA and AURA2) in patients with locally advanced or metastatic disease, osimertinib demonstrated clinical activity in patients with *EGFR* exon 20 p.T790M, supporting its use in patients who have progressed on an *EGFR* tyrosine-kinase inhibitor [22]. Compared to plasma genotyping assays using NGS, the sensitivity and specificity for the detection of *EGFR* exon 20 p.T790M were 91.5% and 91.1%, respectively, using the cobas^®^ mutation test. These were in contrast to tissue genotyping, where sensitivity and specificity were lower, at 61.4% and 78.6%, respectively [19]. This discordance was hypothesized to be due, at least in part, to the heterogeneity of resistance mutations present in tumor tissue.

Recently, circulating tumor cells (CTCs) and inflammatory markers such as C-reactive protein, neutrophil-to-lymphocyte ratio (NLR), platelet-to-lymphocyte ratio (PLR), lymphocyte-to-monocyte ratio (LMR), lactate dehydrogenase (LDH) and the lung immune prognostic index (LIPI) have gained considerable interest as potential markers in the diagnosis, prognosis and monitoring of treatment response in cancer, including lung cancer [23,24]. Evidence is emerging to suggest the possible clinical relevance of CTC detection and enumeration using liquid biopsies and peripheral pro-inflammatory status, which has been associated with worse outcomes in patients with different cancer types [25,26].

In this study, patients with *EGFR*-mutated NSCLC were assessed for *EGFR*-sensitizing and *EGFR* exon 20 p.T790M resistance mutations using longitudinal samples of K2EDTA whole blood (standard of care) and blood collection tubes with a novel stabilization system for comparison (Roche). The cobas^®^ *EGFR* mutation test v2 was used as a companion diagnostic in parallel with two commercially available next generation sequencing (NGS)-based assays that are currently employed in molecular cancer diagnostics in the clinical setting. We hypothesized that CTCs and inflammatory markers (using NLR and dNLR) are surrogates of response to *EGFR*-targeted therapy that may be used as biomarkers and complement the use of circulating tumor DNA (ctDNA) in the diagnosis and prognosis of NSCLC patients.

## 2. Materials and Methods

### 2.1. Patient Recruitment and Sample Collection

**Figure 1:**
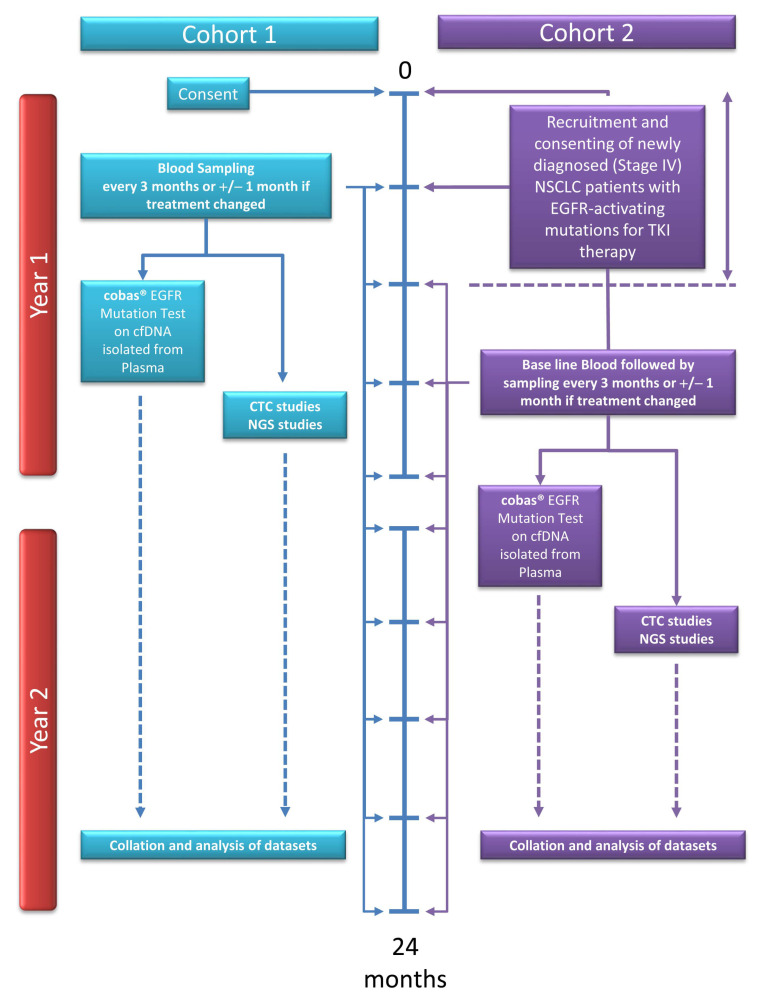
*EGFR* study design. A total of 21 patients with a confirmed tissue diagnosis of epidermal growth factor receptor (*EGFR*) mutated NSCLC were consented and enrolled on this two-year longitudinal study involving three participating Irish hospitals. Patients were stratified into one of two cohorts based on their treatment status: cohort 1 patients were consented during treatment with *EGFR* tyrosine kinase inhibitor (TKI) therapy, while cohort 2 patients were consented at diagnosis, prior to treatment. Liquid biopsy was collected every three months over two years. Plasma genotyping using cell-free DNA (cfDNA), circulating tumor cells (CTCs) and inflammatory markers derived from liquid biopsy at each time-point were examined and correlated with clinical outcomes.

Patients with *EGFR*-mutated NSCLC were recruited prospectively from Medical Oncology departments across three Irish hospitals: St James’s Hospital Dublin, University Hospital Limerick and Midlands Regional Hospital. Following informed consent, patients were recruited into one of two cohorts: cohort 1 (*n* = 9) included those currently receiving *EGFR* TKI therapy at the time of accrual, and cohort 2 (*n* = 12), comprised of newly diagnosed treatment-naïve *EGFR* patients (Figure 1). Whole blood samples were collected longitudinally in 9 mL and 6 mL K2EDTA blood tubes (Cruinn Diagnostics Ltd., Ireland & Becton Dickinson, UK) and Roche CE-IVD cell-free DNA (cfDNA) collection tubes (Roche Molecular Systems, Pleasanton, CA, USA) at baseline and every three months for two years. Blood tubes were mixed by gentle inversion immediately following blood withdrawal. Blood plasma was collected by centrifugation at 3000 rpm for 10 min (EDTA blood tubes) or 1600× *g* for 10 min (cell-free DNA blood tubes) at 48 h and 72 h post-collection per manufacturer’s instructions (Roche Molecular Systems, Pleasanton, CA, USA). All bloods were processed within 4 h of collection. This study was ethically approved by the Tallaght University Hospital and St James’s University Hospital Joint Research Ethics Committee (Ref. No. 041018/8804) and in line with GDPR and Health Research Regulations 2018.

### 2.2. DNA Extraction

Cell-free DNA was extracted from plasma samples using the cobas^®^ cfDNA sample preparation kit (Roche Molecular Systems, Pleasanton, CA, USA) according to manufacturer’s instructions. In brief, 2 mL plasma was diluted in proteinase K and DNA binding buffer, mixed by gentle inversion and incubated for 30 min at room temperature. Following the digestion of DNases, unbound substances such as salts, proteins and other cellular impurities were removed by centrifugation. Bound DNA was washed and subsequently eluted from glass fibre filters. cfDNA was eluted (80 µL) by centrifugation at 8000× *g* for 1 min and stored at –20 °C.

### 2.3. Real-Time RT-PCR

The cobas^®^ *EGFR* mutation test v2 (Roche Molecular Systems, Pleasanton, CA, USA) is a real-time PCR test that can detect 42 mutations in exons 18, 19, 20 and 21 of the *EGFR* gene, including *EGFR* exon 20 p.T790M. PCR amplification was performed according to manufacturer’s instructions. Three working master mixes were prepared, to which magnesium acetate was added. For each master mix, 25 µL was added to each reaction well of a 0.3 mL AD-plate followed by 25 µL of cfDNA. *EGFR* mutant and negative controls (25 µL) were included in all PCR reactions. Plates were spun at 1000 rpm for 1 min and run on a cobas^®^ z 480 real-time PCR analyzer using cobas^®^ 4800 software v2.0.

### 2.4. Circulating Tumor Cell Isolation

CTCs were isolated from K2EDTA blood tubes using ScreenCell^®^ Cyto kits (ScreenCell, Sarcelles, France) for each patient at baseline and every three months during the course of the study. Cells were isolated on the basis of size exclusion technology on a microporous membrane filter and a vacuum tube, allowing leucocytes to pass through the 7.5 μm filter pores, while trapping the larger and less deformable CTCs on the filter, avoiding any bias induced by epithelial cellular adhesion molecule (EpCAM) antibody capture. Briefly, 4 mL ScreenCell^®^ fixation buffer was added to 3 mL whole blood, inverted gently and allowed to stand for 8 min at room temperature. The whole blood/buffer mix was added to a ScreenCell Cyto device and allowed to filtrate through the upper module and filter. Once the mix had passed through to the lower collection module, the ScreenCell^®^ filter was gently washed in PBS, removed from the device, allowed to dry overnight at room temperature and stored at –20 °C.

### 2.5. Morphological Staining of ScreenCell^®^ Filters

ScreenCell^®^ filter morphology was assessed using May–Grünwald Giemsa staining. Filters were placed into a bath containing May–Grünwald (Sigma Aldrich Ltd., Dublin, Ireland) and incubated for 2 min 30 s with constant agitation, followed by a second incubation in a 1:1 dilution of May-Grünwald:dH_2_O (pH 7) for a further 2 min with agitation. Filters were subsequently incubated in a stain consisting of a 1:10 dilution of Giemsa:dH_2_O (pH 7) for 10 min. Excess stain was removed by washing in dH_2_O (pH 7) for 1 min, and filters were allowed to air dry at room temperature for 20 min. Morphological assessment of stained filters was carried out using an Olympus BX41 light microscope (Olympus Life Science, Waltham, MA, USA). CTCs that were identified, based on morphology, were enumerated and recorded by an independent expert cytopathologist (B.H.). CTCs were defined as cells in the same plane of focus as filter pores whose nucleus was at least twice the diameter of a filter pore, dark blue/purple in colour and with an outline that was well-defined around its entire circumference. These criteria have been used for the morphological identification of CTCs in recently published studies of prostate cancer patients [27].

### 2.6. AVENIO ctDNA Targeted Panel Sequencing

NGS libraries were prepared from plasma-derived cfDNA isolated from six *EGFR* patients using the liquid biopsy AVENIO ctDNA targeted panel (Roche, Branchburg, NJ, USA) to identify and detect aberrations such as single nucleotide variants (SNVs), insertions and deletions (Indels) and fusions from NSCLC tumors. A 17-gene pan-cancer targeted panel was used based on guideline-driven biomarkers aligned with the National Comprehensive Cancer Network (NCCN) and included the following genes: *ALK, APC, BRAF, BRCA1, BRCA2, DPYD, EGFR, ERBB2, KIT, KRAS, MET, NRAS, PDGFRA, RET, ROS1, TP53* and *UGT1A1*. cfDNA was isolated from blood plasma using the AVENIO cfDNA isolation kit and quantified using the Qubit™ dsDNA HS assay kit (Thermo Fisher Scientific, Frederick, MD, USA). DNA quality was assessed on High Sensitivity DNA Chips (Agilent Technologies, Santa Clara, CA, USA) using a 2100 Bioanalyzer (Agilent Technologies Germany GmbH, Walbronn, Germany). Input cfDNA (15–50 ng) was used for subsequent library preparation. Samples were prepared for ligation using a PCR master mix and incubated on a thermal cycler at 20 °C for 30 min followed by 65 °C for 30 min. A unique sample adaptor was added to each sample to allow for highly efficient molecular barcoding, ligated in a master mix containing DNA ligase and incubated overnight at 16 °C. Following post-ligation clean-up using clean-up beads and ethanol (80%), the adaptor-ligated DNA samples were amplified by PCR amplification and post-PCR products cleaned as per manufacturer’s instructions. Prior to enrichment, libraries were quantified (Qubit) and DNA quality was assessed (Bioanalyzer). Good quality cfDNA was indicated where no or minimal high molecular weight genomic DNA (>2000 bp) was present. Enrichment of genes from ligated cfDNA was carried out by hybridizing biotinylated enhancing oligos targeting the AVENIO ctDNA panel regions bound to the DNA at 47 °C overnight. These are enriched for blocking repetitive DNA sequences and used to block non-specific hybridization of probes, thereby increasing specificity. Dynabeads M-270 streptavidin were prepared prior to the hybridization clean-up and washing of beads. Amplification of the enriched ctDNA samples was completed on a thermocycler for 15 cycles of 98 °C for 15 s, 60 °C for 30 s and 72 °C for 1 min. Post-capture PCR products were cleaned using clean-up beads, and the final enriched library was quality assessed and quantified. Pooled libraries were diluted to 4 nM and denatured. A 15% PhiX spike-in was used as a sequencing control in line with manufacturer’s guidelines (Illumina, San Diego, CA, USA). Enriched libraries were sequenced on the NextSeq 500 using the High Output Kit v2 (300 cycles) with a median unique target sequence coverage of 7600× (range 2013×–12467×). NGS run metrics included a total number of 510 M read pairs in the lane passing the Illumina filter on the sequencer. The percentage of all read bases with a quality score of at least 30, corresponding to a probability of an error in base calling of 1/1000, was 82.83%. The percentage of reads aligned to the Illumina PhiX sequencing control was 14.33% (typical range: 10–15%). Automated processing of raw data and subsequent analysis was performed using AVENIO ctDNA analysis software version 1.2 (Roche Sequencing Solutions, Inc., Pleasanton, CA, USA). A CSV template was uploaded to the Roche server, which contained adapter details, enabling the de-multiplexing of samples. The remaining analysis and resulting variant and sample metrics were recorded.

### 2.7. Oncomine™ Lung cfDNA Assay

Plasma samples were processed for cfDNA extraction using the cobas^®^ sample cfDNA sample preparation kit (Roche Molecular Systems, Pleasanton, CA, USA) followed by quantification using the Qubit™ dsDNA HS assay. Genomic profiling of samples by targeted NGS was performed using the Oncomine™ Lung cfDNA assay (Thermo Fisher Scientific, Frederick, MD, USA). The assay covers 169 key hotspot mutations across 11 genes and includes *ALK, BRAF, EGFR, ERBB2, KRAS, MAP2K1, MET, NRAS, PIK3CA, ROS1* and *TP53*. In brief, library preparation was performed according to manufacturer’s instructions. cfDNA targets were amplified using a cfDNA library PCR master mix and purified as per manufacturer’s recommendations. Purifications were carried out using AMPure™ XP magnetic beads (Beckman Coulter, Pasadena, CA, USA). Target amplicons were amplified with Tag Sequencing barcodes, and the resulting barcoded library was further purified. Library quantification was performed by qPCR using the Ion Library TaqMan^®^ Quantitation kit (Thermo Fisher Scientific, Frederick, MD, USA) and run on the 7500 Fast Real-Time PCR system (Applied Biosystems, Waltham, MA, USA). Serial dilutions of the *E. coli* DH10B Ion Control Library were used as standards. No template controls (NTC) were also included. Sample barcoded libraries were diluted to a final concentration of 100 pM, pooled together for template preparation on the Ion Chef™ system and loaded onto an Ion 520 chip (Ion 520™ Chip Kit, Thermo Fisher Scientific). The chip was subsequently sequenced on the Ion Chef™ S5 system (Thermo Fisher Scientific, Frederick, MD, USA). Analysis of raw sequencing data was performed using Torrent Suite Software (v5.6). The unaligned BAM (Binary Alignment Map) files generated by the sequencer were mapped against the human reference genome (GRCh37/hg19) using the Torrent Mapping Alignment Program (TMAP) and Ion Torrent Suite™ Software (TSS, version 5.10). The Torrent Variant Caller (TVC, version 5.10) plugin was used for the analysis and annotation of variants using pre-configured parameter settings for the application of liquid biopsy. Analysis, annotation of variants and filtering were performed on the Ion Reporter (v5.6) platform using the Oncomine™ Lung Liquid Biopsy workflow v1.3. The Lung Liquid Biopsy workflow combines the read depth for each region of interest to calculate a limit of detection (LoD). Each sample can have a different LoD depending on read depth and sequence quality. Samples were not deemed to pass quality control unless a LoD of 0.1% was achieved.

### 2.8. Inflammatory Marker Analysis

Complete blood counts and biochemistry (LDH) were obtained for all patients at baseline and at each time-point (every 3 months) throughout the study from the electronic patient records (EPR) at each participating hospital. The NLR, which has previously been demonstrated to play a predictive role in the prognosis of acute and chronic inflammatory processes [28], was measured by dividing the number of neutrophils by the number of lymphocytes. The dNLR was calculated as [absolute neutrophil count]/[white blood cell concentration − absolute neutrophil count] [29,30].

### 2.9. Statistical Analysis

Analysis between groups was carried using student’s *t*-tests or analysis of variance (ANOVA) with Bonferroni post-hoc analysis. The chi-squared test was used to compare categorical variables between groups. Scatter plots with a line of best fit were plotted to explore correlations between continuous variables, with a Pearson’s correlation coefficient used to determine if variables were significantly correlated. Time to progression and overall survival based on *EGFR* exon 20 p.T790M status, CTC numbers and inflammatory markers were calculated using univariate Cox proportional hazards analysis based on median cut-off values for each parameter, and Kaplan Meier curves were plotted to visualize the data. Statistical significance was defined as *p* ≤ 0.05. All data were analysed using R statistical software, version 3.6.2.

## 3. Results

### 3.1. Patient Characteristics

From March 2017 to May 2019, a total of 21 NSCLC patients with a confirmed *EGFR* mutation at biopsy (Oncomine™ NGS, as per standard of care) were consented for participation in this longitudinal study (Table 1). At baseline, the median age of participants was 66 years and was composed largely of females (76.19%) with adenocarcinoma histology (95.24%). At the time of informed consent, none of the participants were current smokers, while former smokers (47.62%) and never smokers (52.38%) accounted for most of the study cohort. While approximately 38% had at least one prior line of treatment with a first-generation *EGFR* TKI, 23.81% of patients had either no previous treatment or two previous lines of treatment with first and second-generation *EGFR* TKIs—erlotinib and afatinib, respectively. The most common *EGFR* sensitizing mutations, exon 19 deletions (61.90%) and L858R (28.57%) were largely represented at baseline in NSCLC tissue biopsies. One patient had both an *EGFR* sensitizing (exon 19 deletion) and resistance mutation (*EGFR* exon 20 p.T790M) at baseline.

### 3.2. Detection of EGFR Mutations in Cell-Free DNA Using Plasma Genotyping

**Figure 2:**
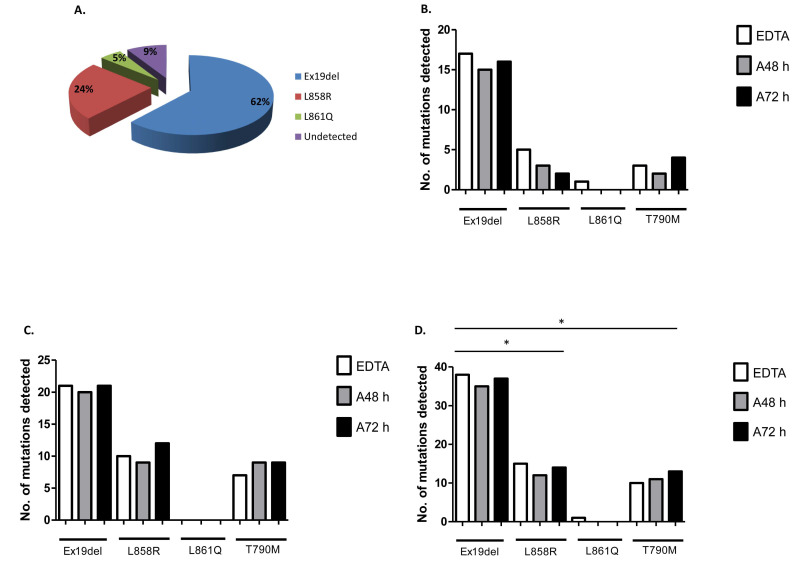
Plasma genotyping of *EGFR* mutations using the cobas^®^ *EGFR* mutation v2 test. Plasma genotyping of *EGFR* mutations using cfDNA from liquid biopsy was carried out using the cobas^®^ *EGFR* mutation v2 test. *EGFR* mutations identified included exon 19 deletions, L858R point mutations and L861Q mutations in exon 21 (**A**). A comparator analysis of *EGFR* sensitizing and resistance mutations (*EGFR* exon 20 p.T790M) in standard EDTA and CE-IVD cell-free DNA blood collection tubes (Roche) was examined in cohort 1 (**B**) and cohort 2 (**C**). *EGFR* mutations across both cohorts combined are also shown (**D**). Statistical analysis was performed using a two-way ANOVA with Bonferroni post-hoc analysis (* *p* < 0.05).

Plasma genotyping of cfDNA from liquid biopsies identified *EGFR* mutations that included deletions in exon 19, in addition to L858R and L861Q substitution mutations in exon 21 at 62%, 24% and 5%, respectively, using the cobas^®^ *EGFR* mutation test (Figure 2A). In a parallel analysis of *EGFR* mutations in cfDNA derived from standard EDTA blood tubes and CE-IVD cell-free DNA collection tubes (Roche), EGFR mutation profiling of cfDNA derived from whole blood from all patients in cohort 1 (Figure 2B) and cohort 2 (Figure 2C) showed only modest differences in the detection of exon 19 deletions between the different blood tubes over time. There was a slight increase in the number of L858R mutations detected in liquid biopsies from cohort 2 relative to cohort 1. In one patient, a L861Q mutation was detected in cfDNA derived from EDTA blood plasma with no mutation detected in plasma cfDNA from 48 h or 72 h cell-free DNA collection tubes. *EGFR* exon 20 p.T790M was detected in blood plasma from all blood collection tubes with only a modest increase in detection found in plasma-derived cfDNA at 72 h. The total numbers of *EGFR* mutations detected in cfDNA from all blood tubes (EDTA and cell-free) across both patient cohorts (cohort 1 and cohort 2) are shown (Figure 2D). There was a significantly greater number of exon 19 deletions detected in plasma cfDNA derived from liquid biopsy using the cobas^®^ *EGFR* mutation test relative to L858R (*p* = 0.0489) and *EGFR* exon 20 p.T790M (*p* = 0.0264). 

**Figure 3:**
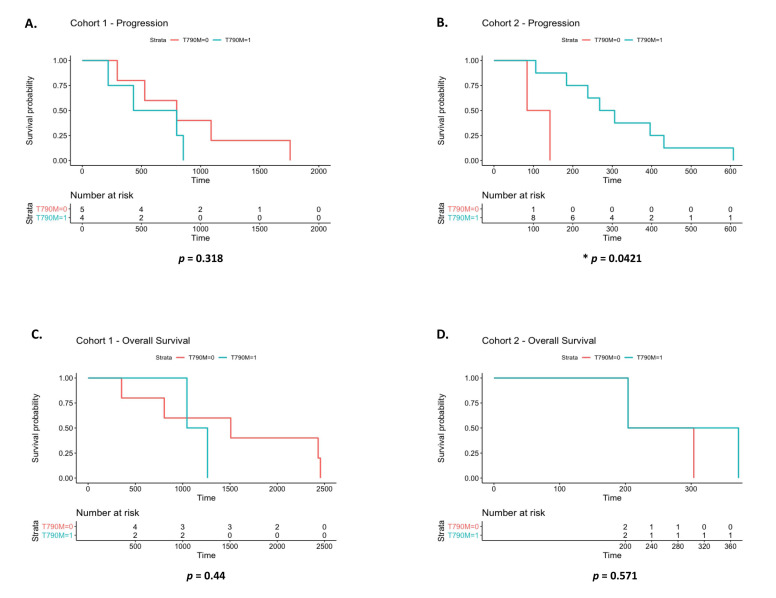

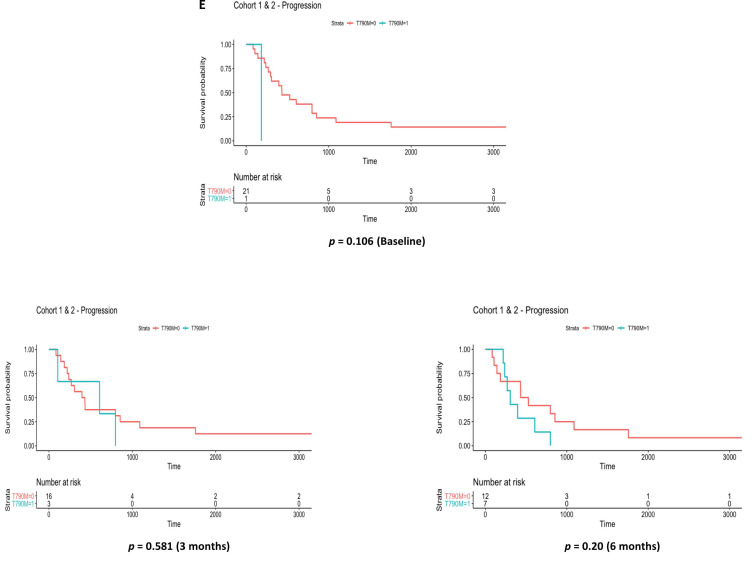
Time to progression and overall survival analysis relative to *EGFR* exon 20 p.T790M detection. The presence or absence of *EGFR* exon 20 p.T790M was correlated with disease progression (**A**,**B**) and overall survival (**C**,**D**) in cohort 1 and cohort 2 at baseline. Time to progression in both cohorts combined was examined at baseline, 3 months and 6 months (**E**). Univariate Cox proportional hazards analysis of time to progression and overall survival was used and Kaplan Meier survival plots represented (* *p* < 0.05).

The emergence of the *EGFR* exon 20 p.T790M resistance mutation was detected in 57% of patients during treatment with *EGFR* TKIs. The presence or absence of this mutation was correlated with disease progression and overall survival at baseline. At baseline (Figure 3A), those in cohort 1 with *EGFR* exon 20 p.T790M progressed more rapidly than those with an *EGFR* sensitizing mutation. This, however, was not statistically significant (*p* = 0.318) and was in contrast to that observed in cohort 2 (Figure 3B), where those with *EGFR* exon 20 p.T790M had a significantly longer progression-free survival (*p* = 0.0421). This finding must be considered with caution, however, as the number of patients with no mutation at baseline was small (*n* = 2). There were no significant differences in overall survival between patients with *EGFR* exon 20 p.T790M and those with an *EGFR* sensitizing mutation in cohort 1 (*p* = 0.44) (Figure 3C) or cohort 2 (*p* = 0.571) (Figure 3D). When both cohorts were combined, no significant differences were found between *EGFR* exon 20 p.T790M and overall survival (Figure S1A–C) or time to progression (Figure S1D–F) at baseline, 3 months or 6 months. Due to the limited number of patients still alive at 2 years, it was not possible to examine the statistical relevance between these parameters at this time-point.

### 3.3. Circulating Tumor Cells as Markers of Disease Outcome

**Figure 4:**
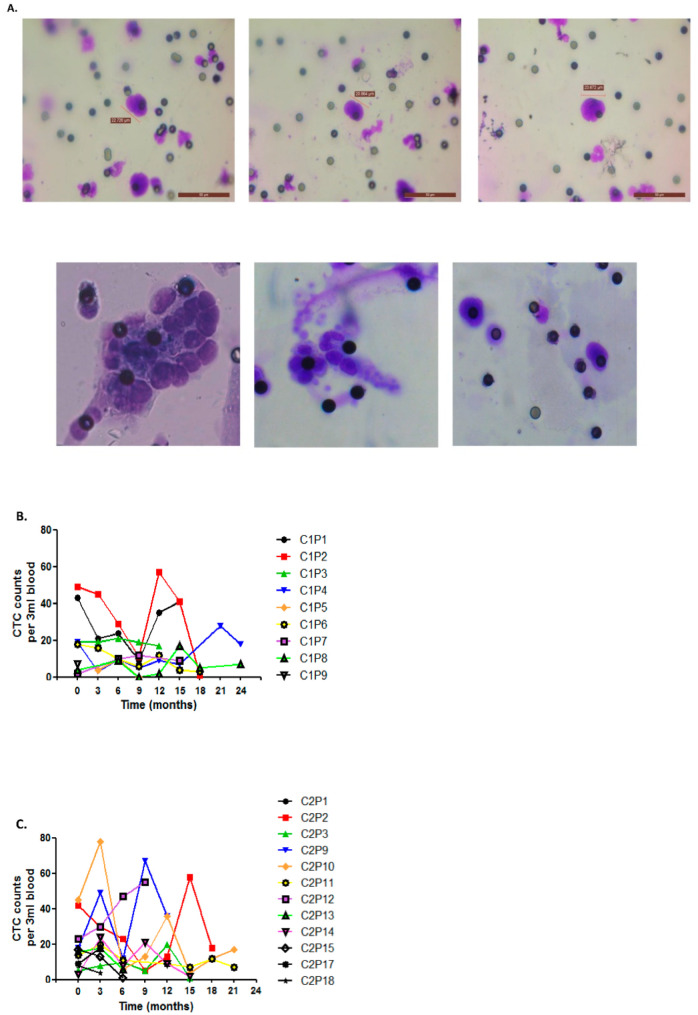
Prospective staining and enumeration of CTCs using ScreenCell^®^ Cyto filters during treatment with EGFR TKIs. CTCs were isolated from liquid biopsies at each 3 month time-point using ScreenCell^®^ Cyto filters based on size exclusion technology. Filters were stained with May–Grünwald Giemsa stain. Morphological assessment and CTC enumeration was carried out by an independent cytopathologist using an Olympus BX41 light microscope. Representative images of CTCs are shown at ×20 magnification (**A**). Longitudinal analysis of CTCs as markers of disease burden and response to EGFR TKI therapy was assessed in *EGFR* cohort 1 (**B**) and cohort 2 (**C**) at baseline and during the 2 year follow-up period.

**Figure 5:**
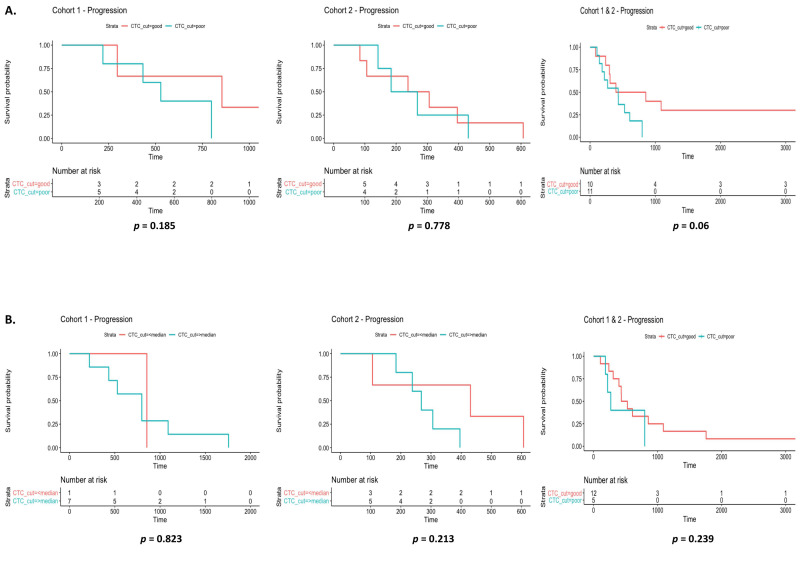

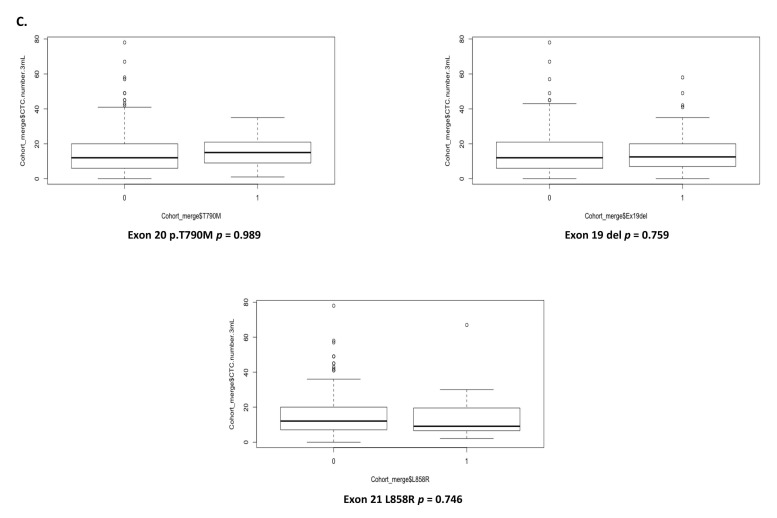
Assessment of CTC numbers and correlation with disease progression. At baseline (**A**) and at 6 months (**B**), the ability of CTC counts to predict disease progression was examined in each cohort individually and combined. No significant differences were observed in time to progression based on CTC counts below and above the median cut-off at baseline (≥16) or at 6 months (≥10). Univariate Cox proportional hazards analysis of time to progression and overall survival was used and Kaplan Meier survival plots represented. Comparison of mean CTC counts by *EGFR* mutation status (exon 20 p.T790M, exon 19del and exon 21 L858R) was examined at baseline in cohorts 1 and 2 combined using student’s *t*-tests (**C**).

**Figure 6:**
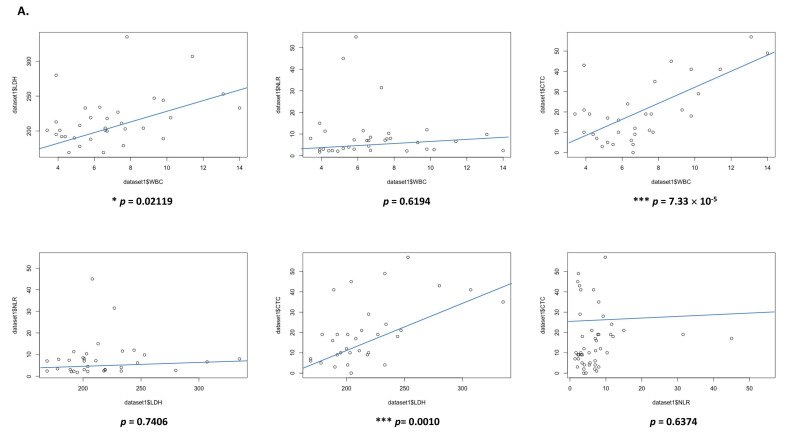

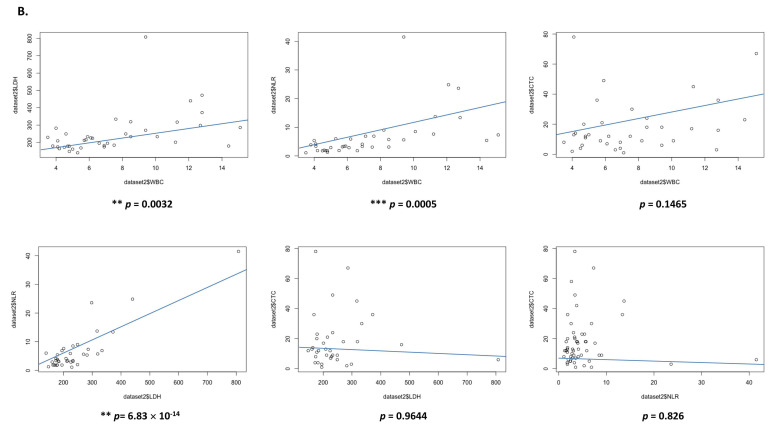
Correlations between systemic inflammatory markers and CTCs. At baseline, correlations between peripheral blood inflammatory markers (WBCs, LDH, NLR, dNLR, CTCs) were examined in cohort 1 (**A**) and cohort 2 (**B**). Scatter plots and Pearson’s correlation coefficients show correlations between continuous blood biomarkers (* *p* < 0.05, ** *p* < 0.01, *** *p* < 0.001).

The detection and isolation of CTCs in the peripheral blood of NSCLC patients remains a challenging strategy in the diagnosis and monitoring of treatment response. It has been reported in advanced lung cancer patients that CTCs are present in the blood at relatively low concentrations. In approximately 1 mL of peripheral whole blood, this typically contains 1–10 CTCs against a background of 10^6^–10^7^ nucleated blood cells and approximately 10^9^ red blood cells [31]. Using size-exclusion technology, CTCs were isolated, stained and enumerated from liquid biopsies (3 mL) at each time-point. These varied in size from 20 to 24 µm (Figure 4A), consistent with those previously reported for CTCs (9–30 µm) [32]. To explore the sequential analysis of CTC counts and their potential indication as a measure of disease burden, CTC numbers were shown to vary considerably between patients at baseline, with transient changes in numbers during treatment with *EGFR* TKIs in cohort 1 (Figure 4B) and cohort 2 (Figure 4C) over the study period of two years. Such alterations in CTC counts at the various time-points may reflect the response to *EGFR* TKIs during treatment. At baseline (Figure 5A) and 6 months (Figure 5B), the ability of CTC counts to predict disease progression was examined in each cohort and combined. No significant differences were observed in time to progression based on CTC counts using median cut-off values at baseline (≥16 per 3 mL blood) and 6 months (≥10 per 3 mL blood). At baseline, however, when both cohorts were combined, a trend towards significance was observed (*p* = 0.06), where patients with high CTC counts had a shorter time to progression. When examined in patients with a resistance mutation or *EGFR* sensitizing mutations at baseline, no significant associations were found between CTCs and *EGFR* exon 20 p.T790M (*p* = 0.989), exon 19 deletions (*p* = 0.759) or exon 21 L858R mutations (*p* = 0.746) (Figure 5C).

### 3.4. EGFR Exon 20 p.T790M Detection by Liquid Biopsy vs. Clinical Progression by CT Imaging

During the two years of the study, the emergence of *EGFR* exon 20 p.T790M was detected in 12 (57%) patients using cfDNA from liquid biopsy during serial blood samping every 3 months. Of these, 4 (33%) were detected in cohort 1, while the remaining 8 (67%) were detected in cohort 2. In order to elucidate the timing of *EGFR* exon 20 p.T790M detection using liquid biopsy relative to clinical progression by radiological computed tomography (CT) imaging, the time interval between these was calculated for each patient where *EGFR* exon 20 p.T790M was detected (Table 2). In both cohorts combined, 67% had an *EGFR* exon 20 p.T790M detected by liquid biopsy prior to a clinical diagnosis of disease progression by CT imaging. The average time interval between the detection of *EGFR* exon 20 p.T790M by liquid biopsy and a radiological CT diagnosis of disease progression was 166.25 days (5.5 months) for both cohorts combined (*p* = 0.066) and 189.5 days (6.23 months) for cohort 2 (*p* = 0.053), where the latter had a greater number of *EGFR* exon 20 p.T790M mutations detected. There was no statistically significant relationship between CTC numbers and time to progression.

### 3.5. Comparative Assessment of NGS ctDNA Analysis Platforms

An assessment of analytical performance in *EGFR* mutation detection was compared between the cobas^®^ *EGFR* mutation test and two next-generation sequencing (NGS) cell-free DNA panels: Oncomine™ lung cfDNA assay and AVENIO ctDNA targeted panel. A preliminary analysis of a small cohort of plasma cfDNA from different blood collection tubes (*n* = 12) that was acquired prospectively during the study compared *EGFR* mutation detection in liquid biopsies using the cobas^®^ *EGFR* mutation test and the Oncomine™ NGS assay (Table 3). These were examined in parallel to the *EGFR* mutation status known for each patient based on the original tissue diagnosis. *EGFR* mutations were more frequently detected in liquid biopsies using the cobas^®^ mutation test compared to those detected using the Oncomine™ lung cfDNA assay across the plasma cfDNA examined. Furthermore, the detection of *EGFR* exon 20 p.T790M was significantly greater (*p* = 0.05) when analysed using the cobas^®^ test compared to those detected using the Oncomine™ assay. In two samples analysed as part of this comparative assessment, no mutations were detected (NMD) using the Oncomine™ panel due to low reads. When *EGFR* mutations detected by liquid biopsy using the Oncomine™ assay were compared to those initially detected by tissue biopsy, there was a significant difference (*p* = 0.017) in the number of *EGFR* mutations detected. It must be noted, however, that the time of sampling of the tissue and liquid biopsy for these patients was not uniform, with liquid biopsies acquired up to several weeks or months following a tissue diagnosis (range 20–31 months for C1; range 1–7 months for C2). As such, these observations must be interpreted accordingly and with caution. In the second part of this analysis of three mutation detection platforms, plasma cfDNA samples were used to assess the detection of *EGFR* mutations in liquid biopsies between the cobas^®^ mutation test and the AVENIO ctDNA targeted panel (Table 4). While there was little or no difference in *EGFR* mutations detected across all samples using the two platforms (*p* = 0.94), compared to mutations detected in the original tissue biopsy, both assays identified *EGFR* exon 20 p.T790M in 33% of patients.

### 3.6. Prognostic Value of Systemic Inflammatory Markers in EGFR Patients

Previous studies in cancer have suggested the importance of the baseline NLR and LDH levels in determining outcomes. Furthermore, the systemic inflammatory status has been reported to correlate with a worse prognosis in patients with lung cancer treated with chemotherapy and, more recently, targeted therapies [33]. We performed an exploratory analysis by evaluating inflammatory markers and survival outcomes in our study cohort. While the NLR is a well-known prognostic factor in NSCLC, the dNLR includes monocytes and other granulocyte subpopulations and may therefore be of more relevance as a prognostic indicator. Correlations between peripheral blood inflammatory markers (neutrophils, WCC, LDH, CTCs, NLR and dNLR) were examined in both cohorts at baseline and in combined cohorts at 3 and 6 months. At baseline, significant positive correlations were found between LDH vs. WBCs (*p* = 0.02119), CTCs vs. WBCs (*p* = 7.33 × 10^−^^5^) and CTCs vs. LDH (*p* = 0.001) in cohort 1 (Figure 6A). Similarly, in cohort 2 (Figure 6B), significant positive correlations were found between LDH vs. WBCs (*p* = 0.003), NLR vs. WBCs (*p* = 0.0005) and NLR vs. LDH (*p* = 6.83 × 10^−^^14^). Of interest, highly significant correlations were observed across all inflammatory markers examined at baseline, 3 months and 6 months (Figure S2).

**Figure 7:**
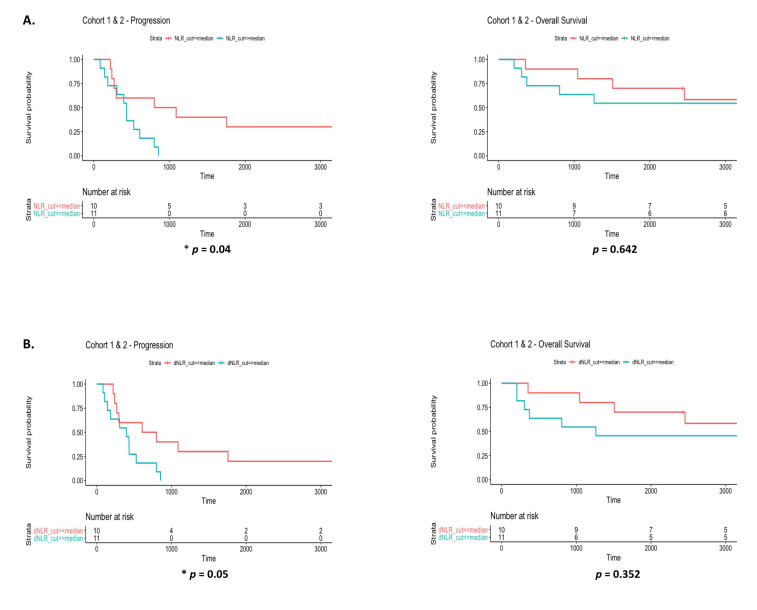

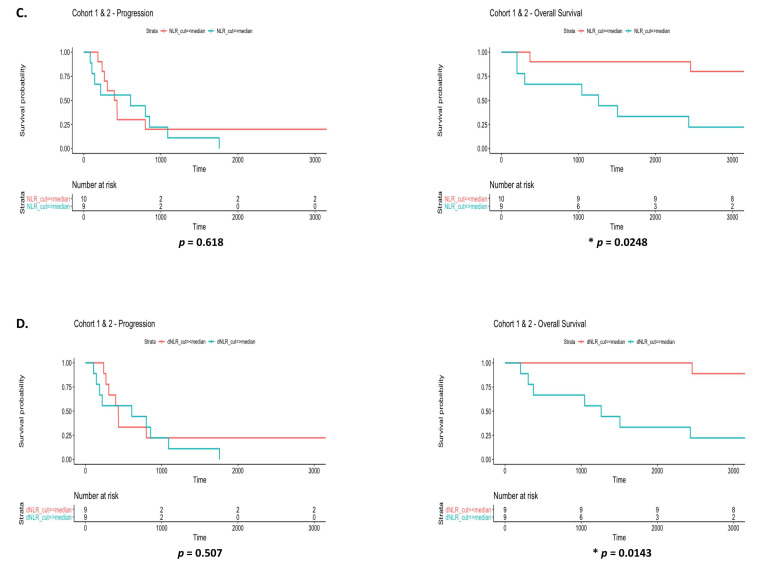

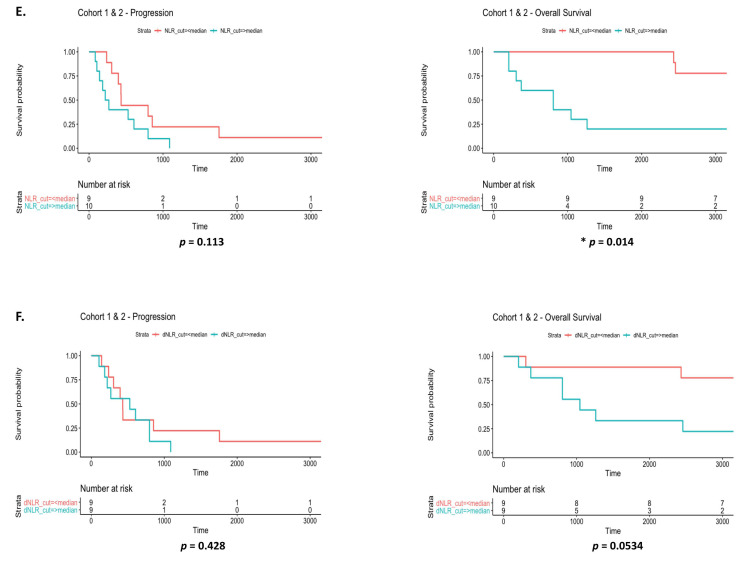
Prognostic significance of the systemic inflammatory markers NLR and dNLR. Systemic inflammatory markers (NLR and dNLR) were correlated with time to progression and overall survival at baseline (**A**,**B**), 3 months (**C**,**D**) and 6 months (**E**,**F**). The median cut-offs for NLR and dNLR at each of the three time-points were ≥5.37 and ≥3, ≥3.42 and ≥2.28, and ≥4.64 and ≥2.17, respectively. Univariate Cox proportional hazards analysis of time to progression and overall survival was used and Kaplan Meier survival plots represented (* *p* < 0.05).

The prognostic significance of the systemic inflammatory markers, NLR and dNLR at baseline, 3 and 6 months was examined. When both cohorts were combined at baseline, high NLR (Figure 7A) (*p* = 0.04) and dNLR (Figure 7B) (*p* = 0.05), above the median cut-off values of ≥5.37 and ≥3, respectively, were significantly associated with a shorter time to progression. No differences were observed in overall survival for NLR (*p* = 0.642) or dNLR (*p* = 0.352). At 3 months, and in contrast to that observed for NLR and dNLR at baseline, high NLR (Figure 7C) (*p* = 0.024) and dNLR (Figure 7D) (*p* = 0.0143) above the median cut-off values of ≥3.42 and ≥2.28, respectively, were significantly associated with a worse survival outcome. No differences were observed in time to progression for NLR (*p* = 0.618) or dNLR (*p* = 0.507). At 6 months, while poor survival outcomes were significantly associated with a high NLR (*p* = 0.014) (Figure 7E), there was a trend towards significance in patients with a high dNLR and worse survival outcome (*p* = 0.0534) (Figure 7F). Median cut-off values for NLR and dNLR at 6 months were ≥4.64 and ≥2.17, respectively. When both NLR and dNLR were correlated with each cohort independently based on time to progression and overall survival, a trend towards a shorter time to progression was observed for a high dNLR (*p* = 0.097) (Figure S3B) at 3 months compared to baseline levels (*p* = 0.665) (Figure S3A), with little or no correlations observed in terms of overall survival at baseline (Figure S3C) or 3 months (Figure S3D). No significant correlations were observed between high NLR and time to progression or survival when examined at baseline and at 3 months.

**Figure 8:**
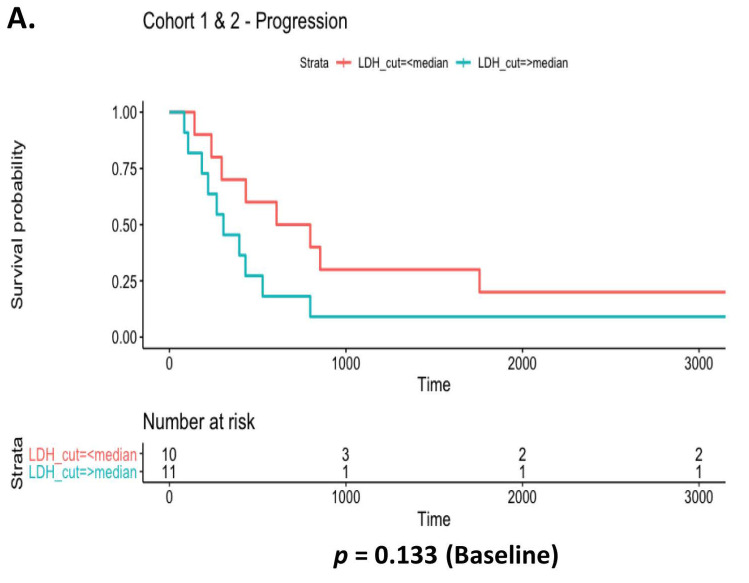

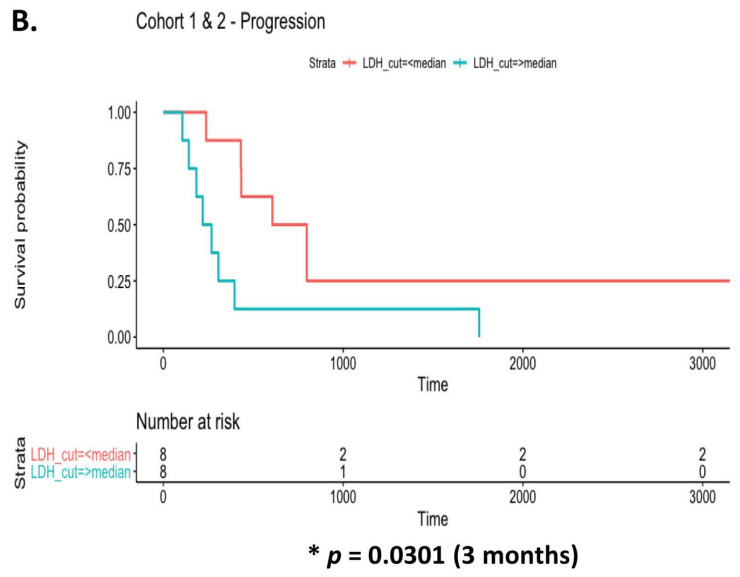
LDH as a prognostic biomarker of disease progression. Relative to LDH levels at baseline (**A**), high LDH levels above the median cut off (≥220.5) were significantly associated with a shorter time to disease progression at 3 months (**B**). The median cut-off for LDH at baseline was ≥229. Univariate Cox proportional hazards analysis of time to progression was used and Kaplan Meier survival plots represented (* *p* < 0.05).

**Figure 9:**
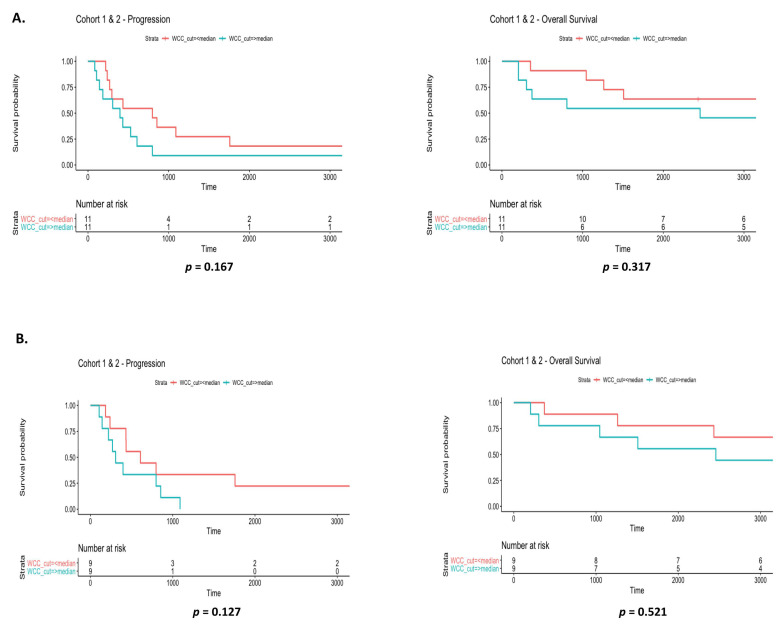

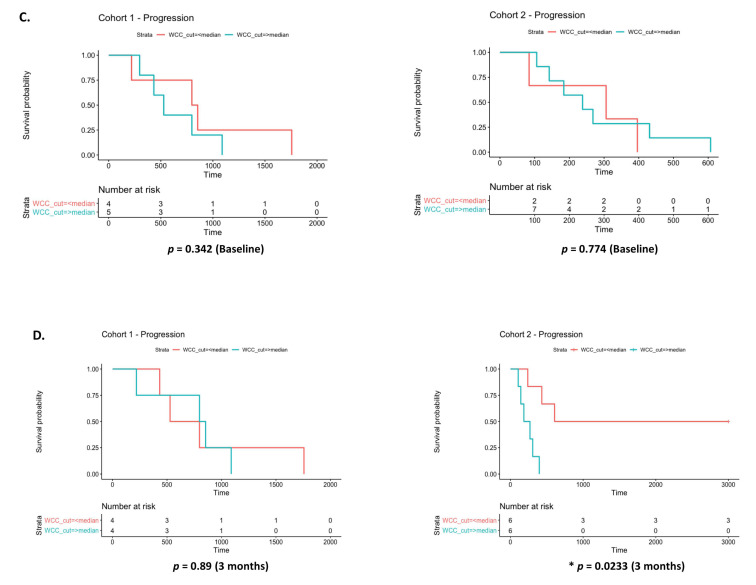
Prognostic significance of WCC in different cohorts. Automated white cell counts were recorded for EGFR patients. Combined cohorts in addition to each cohort alone were examined at baseline and at 3 months for the prognostic significance of white cell counts at baseline and at 3 months, using median cut-off values of ≥9.29 and ≥6.11, respectively. Combined WCC at baseline (**A**) and at 3 months (**B**), had no prognostic significance on time to progression or overall survival. When stratified into respective cohorts (1 and 2), patients in cohort 2 with high WCC had a significantly shorter time to disease progression (**C**,**D**). Univariate Cox proportional hazards analysis of time to progression was used and Kaplan Meier survival plots represented (* *p* < 0.05).

The metabolic and proliferation biomarker, LDH, was examined at baseline (Figure 8A) and at 3 months (Figure 8B). High LDH (IU/L) levels above the median cut-off value of ≥220.5 were significantly associated with a shorter time to disease progression when examined across cohorts at 3 months (*p* = 0.0301) relative to those observed at baseline (*p* = 0.133). Median cut-off value for LDH at 3 months was ≥229. There were no significant correlations between LDH levels and time to progression (Figure S4A) or overall survival (Figure S4B) when cohorts were analysed independently at baseline. Automated white cell counts were recorded for all *EGFR* patients at baseline and at 3 months, and median cut-off values of ≥9.29 and ≥6.11 were determined, respectively. When combined cohorts were examined, WCC at baseline (Figure 9A) had no prognostic value in relation to time to progression (*p* = 0.167) or overall survival (*p* = 0.317). Similar findings were observed at 3 months (Figure 9B). When both cohorts were stratified and examined independently as cohort 1 and cohort 2, those in cohort 2 with a high WCC at 3 months had a significantly shorter time to disease progression (*p* = 0.0233) relative to baseline counts (*p* = 0.774). There were no differences in survival outcomes and WCC when examined at baseline (*p* = 0.317) and at 3 months (*p* = 0.521) (Figure S5).

## 4. Discussion

Recent advances in technologies have allowed for the development of novel plasma genotyping assays for detecting targetable alterations in plasma-derived cell-free DNA using non-invasive strategies, thereby avoiding the inherent risks associated with traditional tissue genotyping [34]. *EGFR* mutations identified in cfDNA isolated from blood have been shown to predict response to the first-generation EGFR TKIs gefitinib and erlotinib [35]. The semi-quantitative cobas^®^ *EGFR* mutation test is one of few FDA-approved plasma genotyping assays currently available. In a previously reported exploratory analysis, two *EGFR* mutation tests were used to examine matched tissue and blood biopsies from three Asian studies (ENSURE, FASTACT-2, ASPIRATION) using first-line erlotinib with similar intent-to-treat populations [36] to inform the clinical utility of *EGFR* mutation testing in blood cfDNA using the cobas^®^ *EGFR* mutation test v2. *EGFR* mutation testing using blood or liquid biopsy demonstrated high specificity and sensitivity with potential utility in the clinical setting that could be used to complement current tissue-based diagnostic approaches. Based on a retrospective analysis of plasma genotyping, the assay has been approved for the detection of *EGFR* exon 20 p.T790M in patients enrolled on the AURA and AURA2 trials of osimertinib [37]. In the current study, and in collaboration with Roche Molecular Diagnostics, the cobas^®^ *EGFR* mutation test (v2) was used for the detection of *EGFR* sensitizing mutations and the emergence of *EGFR* exon 20 p.T790M in serial liquid biopsies from patients with *EGFR*-mutated NSCLC currently receiving *EGFR* TKIs (cohort 1) or in newly diagnosed treated-naïve patients (cohort 2). Plasma genotyping using the cobas^®^ test, for the most part, identified the classical *EGFR*-sensitizing mutations—namely, exon 19 deletions and L858R mutations—in concordance with those identified in the original tissue biopsy. Over the 2 year period of this study, the emergence of *EGFR* exon 20 p.T790M was detected in 57% of patients during treatment with second-generation *EGFR* TKIs. This is in agreement with rates reported in the literature where *EGFR* exon 20 p.T790M accounted for 50–60% of those with acquired resistance to gefitinib or erlotinib [38]. While exon 19 deletions and exon 21 L858R substitutions account for approximately 90% of *EGFR* mutations in NSCLC, patients with these mutations have longer progression-free survival when treated with TKIs compared with chemotherapy. In a literature-based pooled analysis, data from 25 studies involving 1770 patients examined the prevalence of *EGFR* exon 20 p.T790M upon acquired resistance to *EGFR*-TKIs between exon 19 deletions and L858R mutations [39]. Post-resistance *EGFR* exon 20 p.T790M was more frequent in those with exon 19 deletions compared to those with L858R mutations (53% vs. 36%, respectively; OR 1.87; *p* < 0.001) [40]. In the present study, 69% of patients harbouring an exon 19 deletion in their tumor developed *EGFR* exon 20 p.T790M, while in those with an L858R mutation, 50% had a detectable mutation. To elucidate if this observed increase in *EGFR* exon 20 p.T790M in patients harbouring exon 19 deletions is related to decreased sensitivity to *EGFR* TKIs, requires further investigation.

The exon 21 L861Q mutation was detected in one patient receiving *EGFR* TKI therapy. This mutation is the second most frequent uncommon mutation, accounts for approximately 2% of *EGFR*-mutated mutations and, in some cases, can be compounded with other mutations [41]. This patient progressed within 9 months of *EGFR* TKI therapy. In preclinical trials, low efficacy or complete resistance of the L861Q mutation to *EGFR*-TKIs has been demonstrated, suggesting a poor prognostic outcome. Moderate responses in patients harbouring the *EGFR* L861Q mutation have been reported by Yang et al. [42], demonstrating high activity of the *EGFR* TKI, afatinib, with an overall response rate (ORR) of 56.3%, median PFS of 8.2 months and median OS of 17.1 months. During the collection of serial liquid biopsies over the course of this study, a comparator analysis of *EGFR* mutations detected using cfDNA isolated from different blood collections tubes was carried out in parallel. At each three month time-point, liquid biopsy was obtained in standard K2EDTA blood tubes and cell-free DNA collection tubes (Roche) containing a proprietary solution to prevent cell lysis, thereby enabling the detection of plasma-derived cfDNA up to 72 h following blood withdrawal. No significant differences were observed across the different blood collection tubes in the detection of either *EGFR*-sensitizing mutations or *EGFR* exon 20 p.T790M. The use of cell-free blood collection tubes may, however, provide a more appropriate carrier for the collection, stabilization and prolonged transportation of whole blood specimens for cfDNA analysis by preserving nucleated cells during transit and may be used over standard EDTA blood tubes in such cases.

CTCs are characteristically shed from primary or metastatic tumors into the blood circulation and as such have been proposed as a potential marker of response to therapy in several tumor types [43,44,45], including NSCLC [46]. We prospectively evaluated the prognostic value of CTCs from liquid biopsy collected every three months until the end of the study at two years. CTC counts above that of the median cut-off value, predicted a worse outcome with shorter progression-free survival. While this did not appear to be significant in cohort 1 (having already started *EGFR* TKI therapy at the time of consent), our data suggest that CTC counts were an independent prognostic factor in *EGFR* TKI treatment-naïve patients (cohort 2). Those with high CTC counts had a reduced progression-free survival. These findings suggest that changes in CTC counts may be associated with therapeutic response and may be used to monitor patients during treatment with *EGFR* TKIs. Previous studies have reported the progressive monitoring and predictive value of CTCs in *EGFR*-mutated NSCLC treated with first-line *EGFR* TKIs [47]. Compared to those with high CTC counts at baseline, patients with low CTC counts had a markedly longer progression-free survival (hazard ratio = 0.48; *p* < 0.001) and overall survival (hazard ratio = 0.52; *p* = 0.002). Others have shown that baseline CTC counts were an independent predictive factor of progression-free survival and overall survival in patients with NSCLC treated with chemotherapy [48]. In a recent phase II study investigating the correlation between the efficacy of *EGFR*-TKIs and CTC levels in patients with advanced NSCLC, low CTC counts were associated with a significantly better objective response rate and longer progression-free survival [49].

The use of NGS has permitted the parallel sequencing of small DNA fragments for testing a wide range of mutational gene panels [50] and is now recognized as an acceptable sequencing method for mutational testing in lung cancer. Combining liquid biopsy with NGS offers the potential to comprehensively profile tumors for specific genomic alterations, while avoiding invasive tissue biopsies. We assessed the *EGFR* mutation profile of patients using targeted NGS and real-time PCR (cobas^®^ *EGFR* mutation test v2) of liquid biopsy and how these platforms compared to standard tissue biopsy (NGS). Liquid biopsy (cfDNA) was used to examine the *EGFR* mutation status in a small cohort, comparing the qPCR-based cobas^®^ test to the NGS Oncomine™ lung panel. The former platform detected significantly more *EGFR* exon 20 p.T790M, all of which co-occurred with their original *EGFR*-sensitizing mutations. Of interest, while *EGFR* exon 20 p.T790M mutations were found in liquid biopsy using both NGS platforms, no resistance mutation was detected by NGS analysis in the corresponding lung tumor tissue at diagnosis. Tissue and blood samples from these patients were not acquired on the same day. This must be considered when interpreting these differences in *EGFR* exon 20 p.T790M detection between liquid and tissue biopsies. This, however, highlights the importance of the follow-up and monitoring of patients using liquid biopsy, which is not otherwise possible with tissue. While cfDNA constitutes a small fraction of the total cell DNA, the absence of *EGFR* exon 20 p.T790M detection in tissue biopsies may reflect intratumoral heterogeneity, in comparison to liquid biopsy from the same patient, where the shedding of tumor DNA may account for the higher numbers of *EGFR* exon 20 p.T790M mutations detected. It is well established that intratumoral heterogeneity inherently limits the accuracy of a tissue biopsy and capturing a snapshot of the mutational status of a tumor. As such, this can give rise to large variability in results from different sites of biopsy [51]. Alternatively, this may simply be due the infrequency of de novo *EGFR* exon 20 p.T790M. In our analyses, where plasma was collected after the initial tumor biopsy taken as part of routine clinical diagnostics, the discordance observed between the liquid biopsy vs tissue biopsy may be due to tumor evolution within this time period, which in turn may result in altered cfDNA levels in the blood, but not seen in tissues [52]. This timing of sample collection is, however, a limitation of the current study. In a recent systematic review by Esagian et al. [53], a comparison of liquid and tissue-based biopsy analyses by targeted NGS was examined in advanced NSCLC. The authors reported that targeted NGS using liquid biopsy fell short in detecting mutations compared to that using tissue biopsy in NSCLC. Based on these data, the application of NGS in the context of liquid biopsy was unable to substitute a tissue biopsy diagnosis. However, the authors suggested that in the case of *EGFR* testing, their data supported the use of liquid biopsy as a useful tool to complement tissue biopsy in specific clinical settings where tissue sampling or otherwise may be inadequate. The use of different polymerase chain reaction (PCR)-based methodologies, such as allele-specific PCR and droplet digital PCR, have also been studied using liquid biopsy to assess prognosis, treatment response and the emergence of resistance in advanced disease, in addition to comparing results derived from blood plasma to those obtained from tissue biopsy [54,55]. Using a targeted *EGFR* panel offers a cheaper approach with a more rapid turnaround time than NGS and furthermore requires less technical expertise. While it offers less output in terms of multiple genomic alterations, the technique is of value in guiding treatment with *EGFR* TKIs, particularly with osimertinib, based on the presence of *EGFR* exon 20 p.T790M [56].

Systemic inflammation is now recognized as a mechanism of immune resistance in cancer. It has been shown to promote cancer growth and metastasis through its activation of oncogenic signalling pathways [57]. Furthermore, a peripheral pro-inflammatory status has been associated with worse outcomes, particularly in patients with early-stage lung cancer [58] and in advanced stage disease, where a negative impact of this inflammatory environment has been reported during treatment with platinum chemotherapy and targeted therapies [59,60]. Peripheral blood markers such as absolute neutrophil count and platelet counts, circulating white blood cells and LDH levels have been studied as inflammatory biomarkers in cancer. The NLR and dNLR have recently been reported as a measure of the inflammatory status in melanoma [30] and NSCLC [29] treated with immune checkpoint inhibitors (ICIs). In the latter study, Mezquita et al. reported the use of the lung immune prognostic index (LIPI) based on a multicentre retrospective study. This comprised of a test (*n* = 161) and validation set (*n* = 305) of patients treated with programmed death 1/programmed death ligand 1(PD-1/PD-L1) inhibitors across eight different European centres. The study also included a control cohort (*n* = 162) treated with chemotherapy only. This composite index was based on a dNLR of greater than 3 and an LDH level higher than the upper limit of normal which were then used to stratify patients into three risk groups: good, intermediate and poor. The authors showed that pre-treatment LIPI correlated with worse outcomes for patients with NSCLC treated with ICIs, but not for chemotherapy. Elevated dNLR was found in 35% of NSCLC patients (*n* = 466) compared to 22.5% reported by Ferruci et al. [30] in melanoma patients (*n* = 720) treated with ipilimumab. Other studies [61] have shown that LIPI is an independent prognostic factor for chemotherapy in wild-type *EGFR* patients and for *EGFR*-TKI therapy in *EGFR*-mutated subsets, but not for squamous cell carcinoma. While LIPI was not possible in our study due to limited patient numbers, these findings suggest that LIPI may be a useful biomarker for chemotherapy and *EGFR*-TKI therapy in specific subsets of NSCLC and highlights the need to evaluate such biomarkers based on histological and genetic subtypes. In the current EGFR study, pre-treatment dNLR levels (>3) were elevated in 60% of newly diagnosed patients. After 3 months of treatment with *EGFR* TKI therapy, dNLR levels remained increased in only 8%, while at 6 months, this systemic pro-inflammatory state was shown to be further elevated in 30% of the study cohort. Furthermore, these elevated increases in dNLR were mirrored by a decreased progression-free survival and shorter overall survival observed in this *EGFR* patient population.

Another classic inflammatory marker, LDH, has also been widely studied in cancer and has been found to be associated with shorter survival in lung cancer [62,63]. In melanoma, Diem et al. [64] reported the use of LDH in predicting early response or disease progression in patients with advanced melanoma during treatment with anti-PD1 therapy. Patients with a relative increase in LDH of >10% from baseline had a significantly shorter overall survival compared to those with a change of ≤10% (4.3 vs. 15.7 months). When combined, we observed a significantly reduced progression-free survival in *EGFR* patients based on LDH values above the median cut-off value at 3 months. While there was a similar trend observed at baseline for progression-free survival and overall survival, these were not statistically significant.

One of the main limitations of the current study is the small number of patients included in this prospective, longitudinal analysis of patients with EGFR-mutated NSCLC. This can be attributed to (a) the low prevalence of EGFR mutations (~11%) in Irish patients and (b) the short time frame for completion of the study (2 years). We attempted to address this by extending recruitment to medical oncology departments from three different hospitals in Ireland to increase patient numbers within the study period. Building on the results described for this limited number of patients, future studies are warranted in a larger cohort of EGFR patients and may require cross-border/international collaborations to further increase the statistical power for this oncogene-driven subset of NSCLC patients. Larger studies are needed to identify optimal threshold cut-off values for CTCs and immune markers examined in the current study.

## 5. Conclusions

In the current study, we show that longitudinal monitoring using plasma-derived cfDNA may be used to detect *EGFR* sensitizing mutations and monitor the emergence of EGFR resistance mutations in patients with a confirmed histological diagnosis of NSCLC. Targeted NGS analysis using cfDNA has significant potential as a highly accurate diagnostic platform for detecting the presence of actionable mutations in lung cancer and for patient stratification as part of clinical trials. Our data also suggest that CTCs may be used as a surrogate marker to monitor treatment response. Furthermore, peripheral blood inflammatory markers hold potential as prognostic markers in NSCLC and may also be used for stratification or enrichment. Moreover, they have the potential to provide valuable information on the prognosis prior to and during treatment and in the risk–benefit ratio that is required when deciding on the best treatment approach. While this study was carried out in a cohort of patients with advanced disease, a future role for liquid biopsy in the early-stage setting is warranted in light of the recent FDA approval of osimertinib as an adjuvant treatment in surgically resected patients with exon 19 deletions or exon 21 L858R mutations.

## Figures and Tables

**Table 1:** Patient demographics and characteristics.

Patient Baseline Characteristics		
Characteristics	Number of Patients	Percentage (%)
**Age, years**		
Median (Range)		66 (40−85)
**Gender**		
Male	5	23.81
Female	16	76.19
**Smoking status**		
Current	0	0
Former smokers	10	47.62
Never smokers	11	52.38
**Histology**		
Adenocarcinoma	20	95.24
Squamous cell carcinoma	1	4.76
**Alive or Dead**		
Alive	10	47.62
Dead	11	52.38
**ECOG status**		
0	3	14.29
1	12	57.14
2	5	23.81
3	1	4.76
**Previous lines of treatment**		
0	5	23.81
1	8	38.10
2	5	23.81
3	1	4.76
4	2	9.52

Consented patients (*n* = 21) with a pathologically confirmed *EGFR* mutation were recruited prospectively from Medical Oncology departments across three participating Irish hospitals: St James’s Hospital Dublin, University Hospital Limerick and Midlands Regional Hospital Tullamore.

**Table 2:** Time analysis of *EGFR* exon 20 p.T790M detection by liquid biopsy and radiological detection of disease progression by CT imaging.

Study ID	Time Difference (Days) between T790M Detection & Disease Progression
C1P1-02	545
C1P3-01	−16
C1P4-04	97
C1P8-05	−177
C2P2-02	−66
C2P3-02	−201
C2P9-00	−203
C2P10-05	40
C2P11-02	−33
C2P13-01	4
C2P14-02	−153
C2P15-01	−481

A comparison between the time of *EGFR* exon 20 p.T790M detection by serial liquid biopsy (cobas^®^ *EGFR* mutation v2 assay) and detection of disease progression by CT imaging was carried out across all patients (*n* = 12) in whom *EGFR* exon 20 p.T790M was detected during the 2 year period of the study. While not statistically significant, there was an emerging trend towards earlier detection of *EGFR* exon 20 p.T790M using serial liquid biopsy compared to radiological detection of disease progression by CT. Positive and negative numbers indicate the difference (number of days) after which *EGFR* exon 20 p.T790M was detected by liquid biopsy post- and pre-CT imaging (disease progression), respectively. Statistical analysis was carried out using a one-sample student *t*-test.

**Table 3:** Assessment of *EGFR* mutation detection using NGS and cobas^®^ *EGFR* mutation v2 assay.

	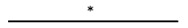		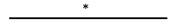	
Study ID	Tissue Biopsyy (NGS)	Liquid Biopsyy (Oncomine™ NGS)		Liquid Biopsyy (cobas^®^)
C1P6-00-E	Ex19del	Ex19del		Ex19del
C1P6-00-A48	Ex19del	NMD		Ex19del
C1P6-00-A72	Ex19del	Ex19del		Ex19del
C1P2-00-E	Ex19del	NMD		NMD
C1P2-00-A48	Ex19del	NMD		Ex19del
C1P2-00-A72	Ex19del	NMD		NMD
C2P2-02-A48	Ex19del	Ex19del, T790M		Ex19del, T790M
C2P13-01-A72	Ex19del	Ex19del		Ex19del, T790M
C2P2090662-E	Ex19del	Ex19del		Ex19del, T790M
C2P12-00-A48	L858R	L858R		L858R
C2P9-00-A48	L858R	NMD*		L858R, T790M
C1P7-00-A48	L858R	NMD*		NMD

NMD*: No mutation detected/low reads. An assessment of analytical performance for the detection of EGFR sensitizing and *EGFR* exon 20 p.T790M mutations was compared between tissue and liquid biopsy using the NGS Oncomine™ lung cfDNA assay and the cobas^®^ *EGFR* mutation v2 test (*n* = 12). A chi-squared test was used to determine whether there were differences in mutation detection between liquid (Oncomine™) vs. liquid (cobas^®^) biopsy and between liquid (Oncomine™) vs. tissue biopsy (* *p* < 0.05).

**Table 4:** Comparative assessment of the AVENIO ctDNA targeted NGS panel and the cobas^®^ *EGFR* mutation v2 test.

		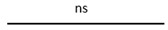	
Study ID	Tissue Biopsy (NGS)	Liquid Biopsyy (Avenio NGS)	Liquid Biopsy (cobas^®^)
C1P9-00-E	Ex19del	Ex19del	L861Q
C1P3-04-A48	Ex19del	Ex19del	Ex19del
C1P4-04-E	Ex19del	Ex19del, T790M	Ex19del, T790M
C2P9-03-E	L858R	L858R	L858R
C1P1-04-E	Ex19del	Ex19del, T790M	Ex19del, T790M
C1P6-04-A48	Ex19del	Ex19del	NMD

Plasma-derived cfDNA was isolated from liquid biopsy (*n* = 6). *EGFR* mutation analysis was compared between tissue and liquid biopsy using the AVENIO ctDNA targeted NGS panel and the cobas^®^ *EGFR* mutation v2 test. A chi-squared test was used to determine statistical significance and whether there were differences in mutation detection between liquid (AVENIO) vs. liquid (cobas^®^) biopsy and between liquid (AVENIO) vs. tissue biopsy (*p* = 0.94).

## Data Availability

These data are not publicly available as this is a study involving human research participants. The data presented in the study are available on request from the corresponding author.

## Supplementary Materials

The following supporting information can be downloaded at: https://www.mdpi.com/article/10.3390/diagnostics12102360/s1, Figure S1: Time to progression and overall survival analysis relative to EGFR exon 20 p.T790M detection; Figure S2: Correlations between systemic inflammatory biomarkers; Figure S3: Prognostic significance of the systemic inflammatory marker, dNLR; Figure S4: LDH as a prognostic biomarker of disease progression and overall survival; Figure S5: Prognostic significance of WCC and overall survival.
