# Supplementary figures and images for: Liquid Biopsy: A Multi-Parametric Analysis of Mutation Status, Circulating Tumor Cells and Inflammatory Markers in EGFR-Mutated NSCLC
Source: Diagnostics (Basel). 2022 Sep 29;12(10):2360. doi: 10.3390/diagnostics12102360 (PMC9600124; doi:10.3390/diagnostics12102360)

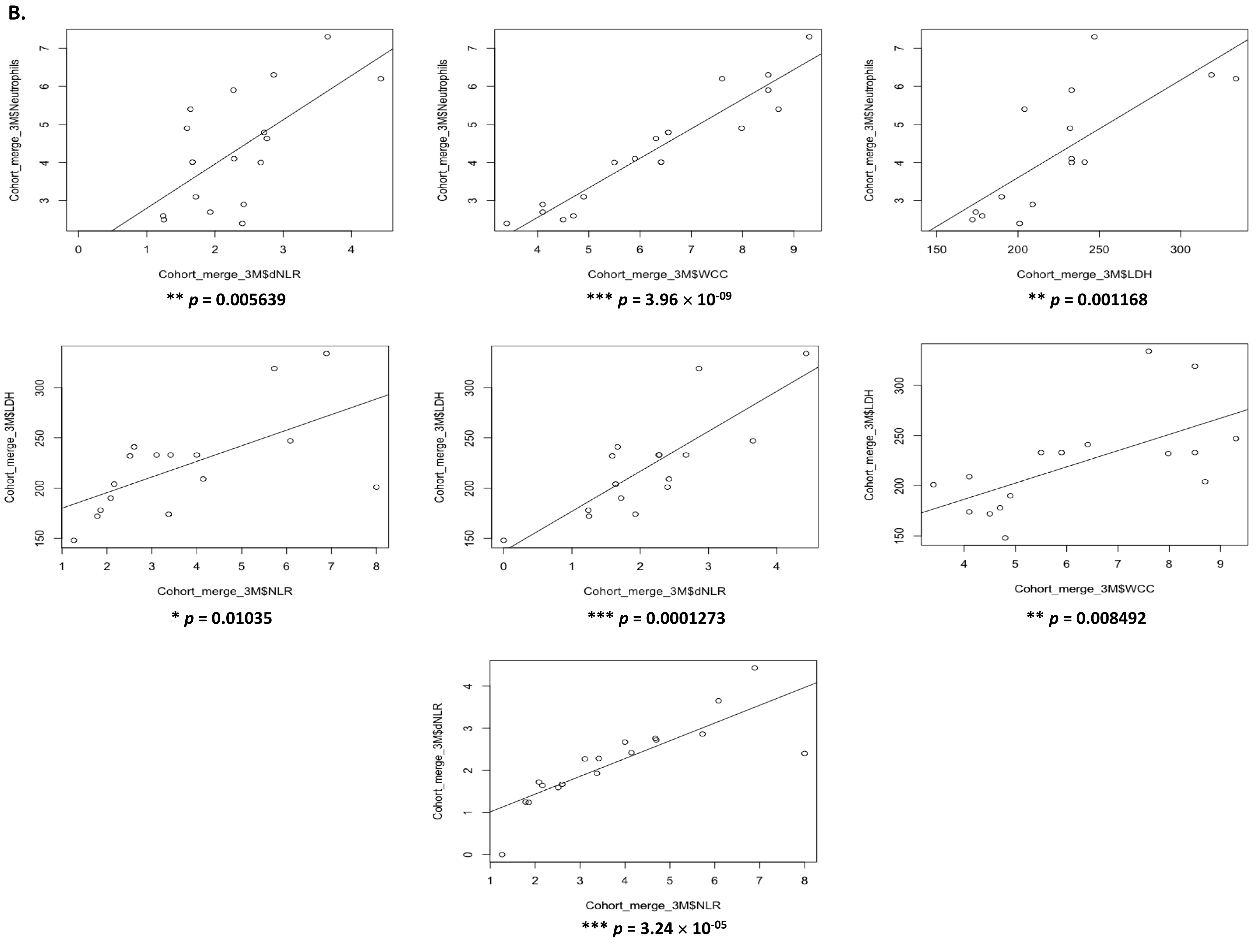

Supplement: Supplementary file 1 [file diagnostics-12-02360-s001.zip › Supplementary Figure S2B_Proof_Corrected.tif]

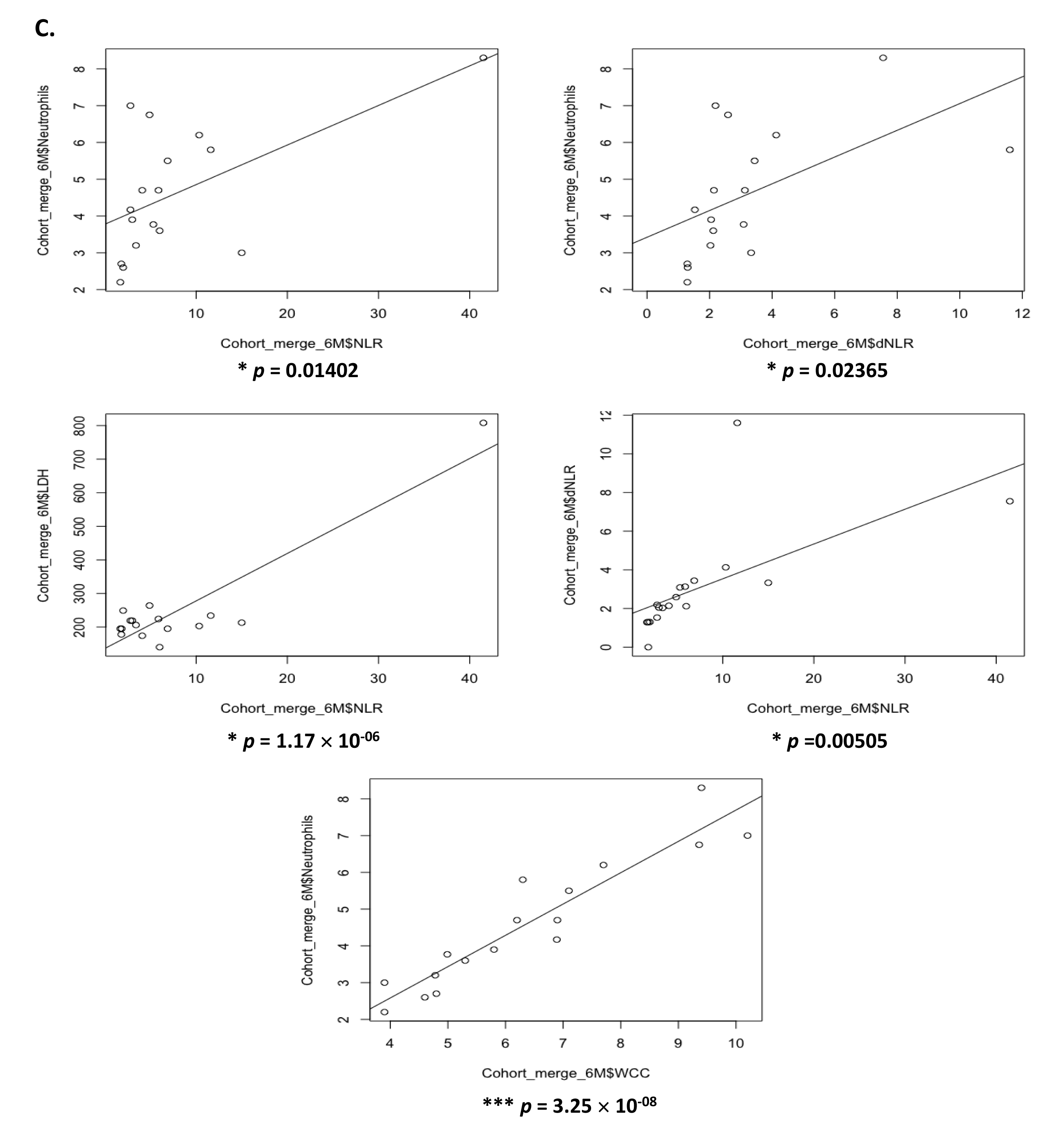

Supplement: Supplementary file 1 [file diagnostics-12-02360-s001.zip › Supplementary Figure S2C_Proof_Corrected.tif]

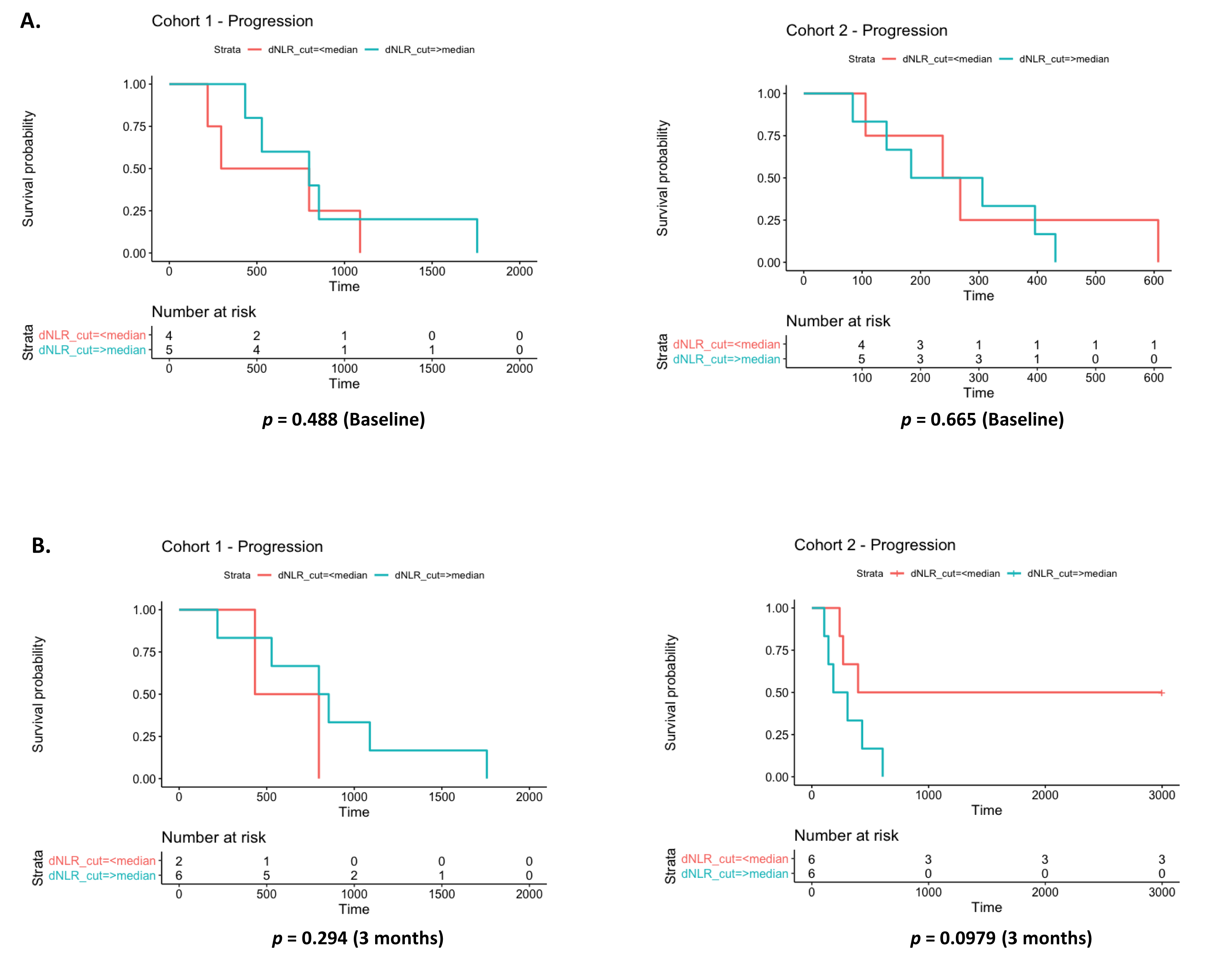

Supplement: Supplementary file 1 [file diagnostics-12-02360-s001.zip › Supplementary Figure S3A_B_Proof_Corrected.tif]

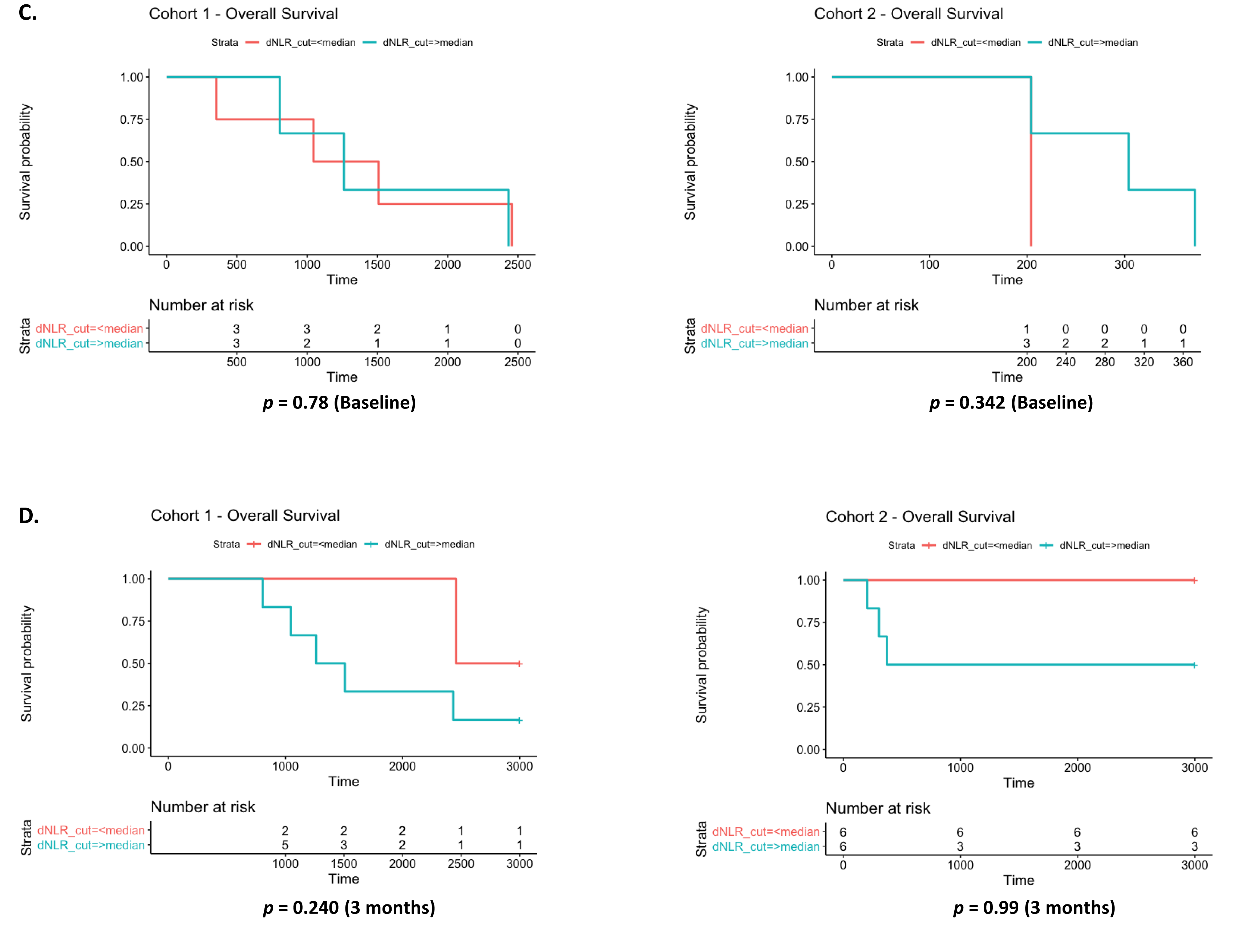

Supplement: Supplementary file 1 [file diagnostics-12-02360-s001.zip › Supplementary Figure S3C_D_Proof_Corrected.tif]

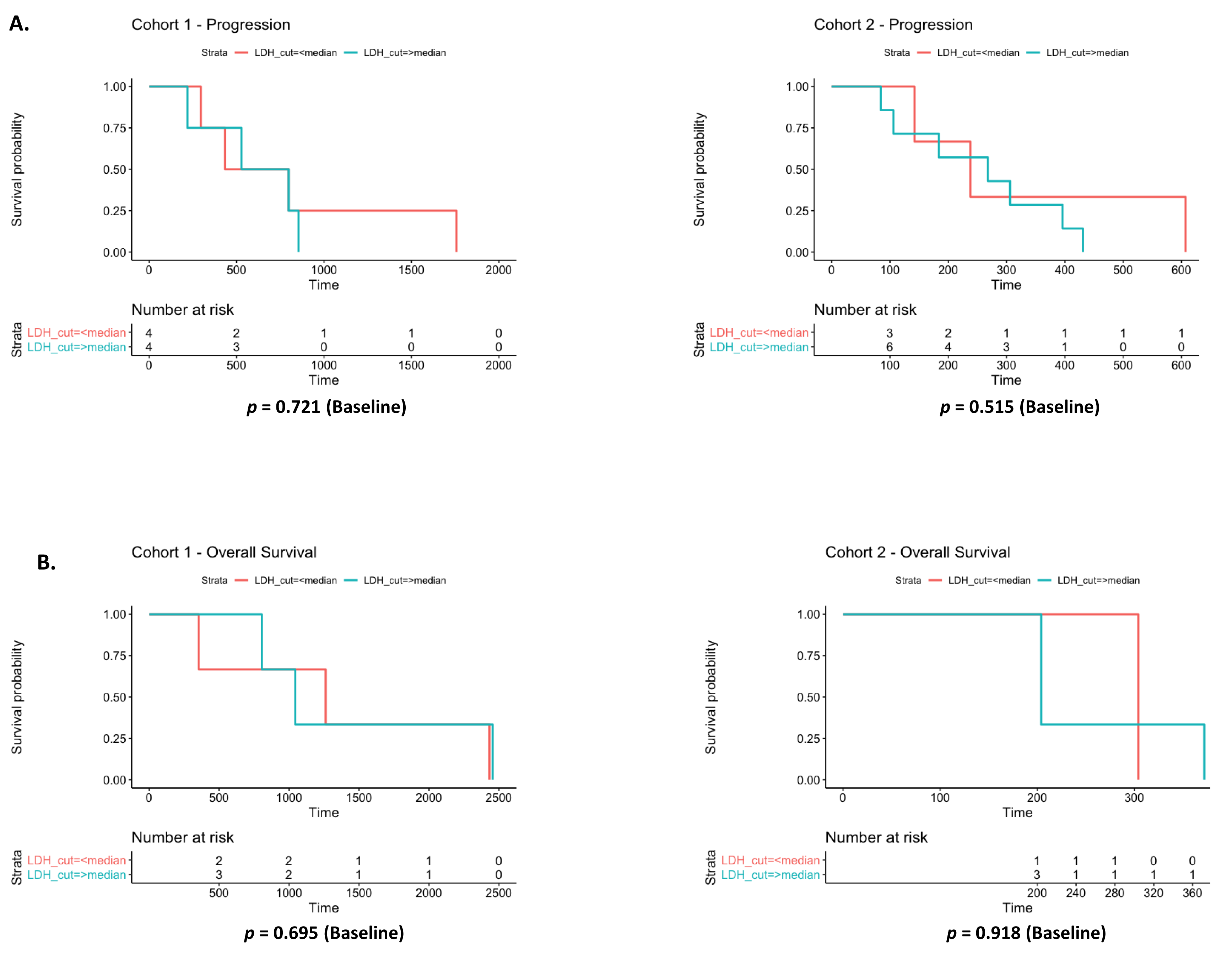

Supplement: Supplementary file 1 [file diagnostics-12-02360-s001.zip › Supplementary Figure S4A_B_Proof_Corrected.tif]

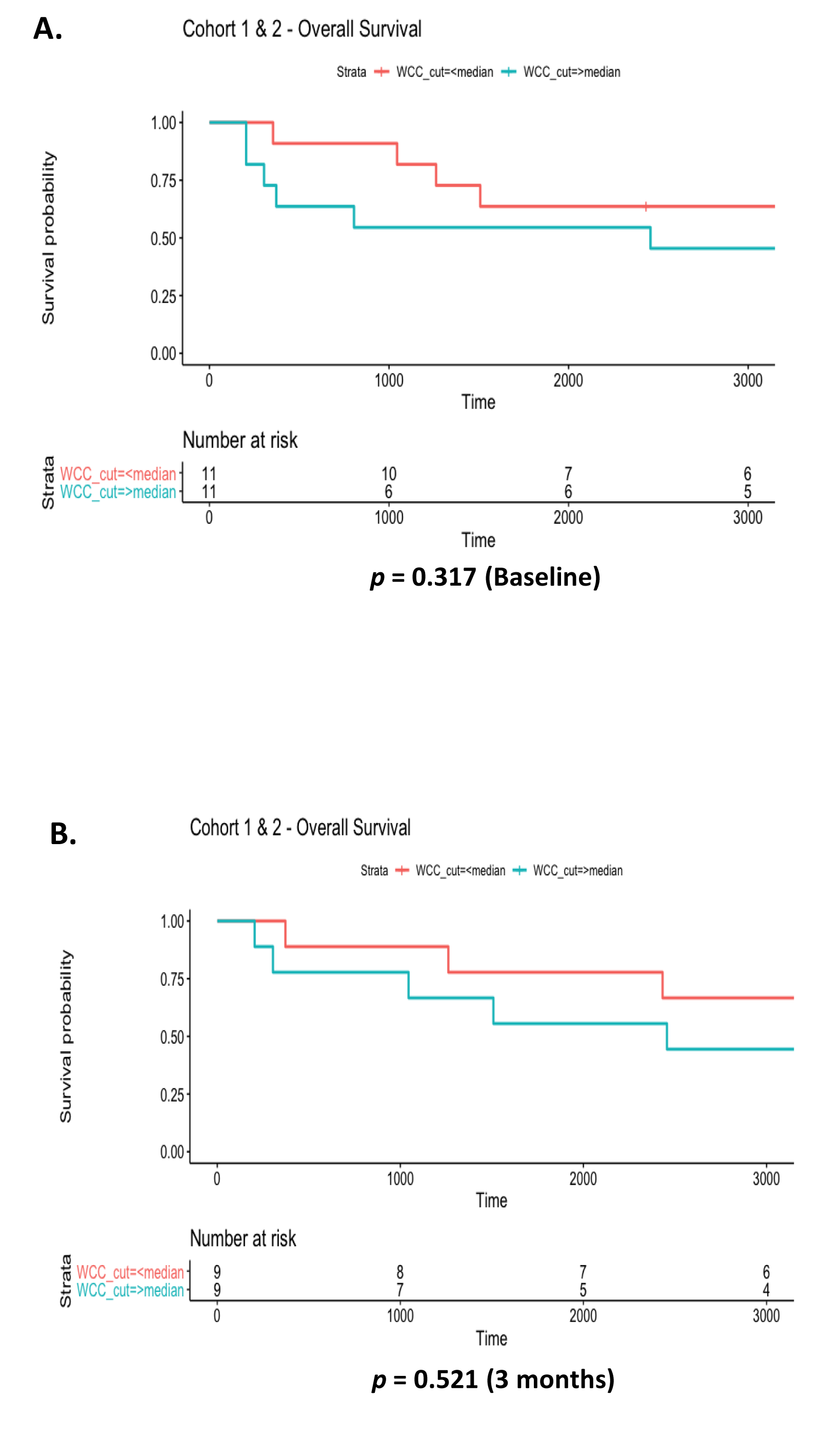

Supplement: Supplementary file 1 [file diagnostics-12-02360-s001.zip › Supplementary Figure S5A_B_Proof_Corrected.tif]

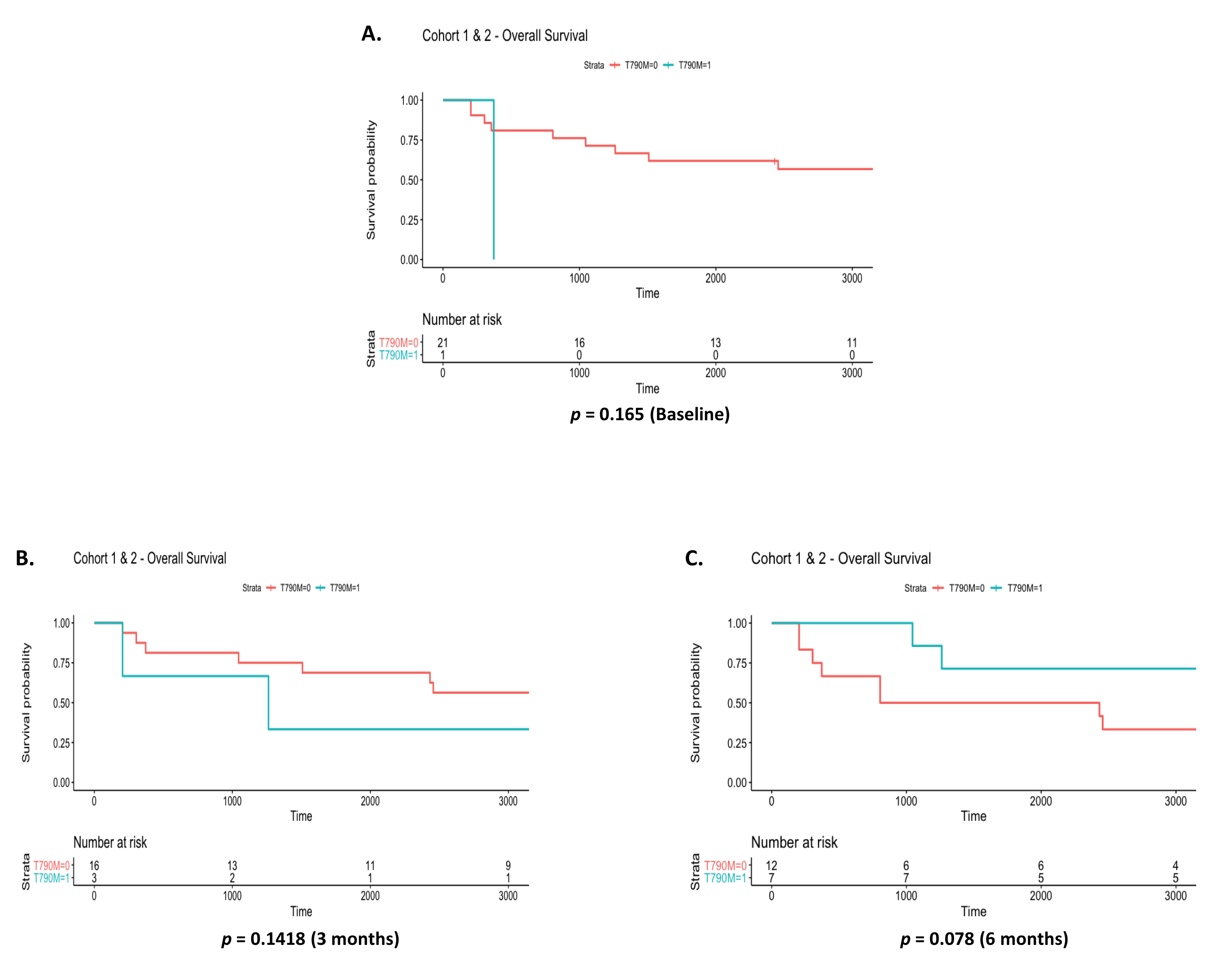

Supplement: Supplementary file 1 [file diagnostics-12-02360-s001.zip › Supplementary Figure S1A_C_Proof_Corrected.tif]

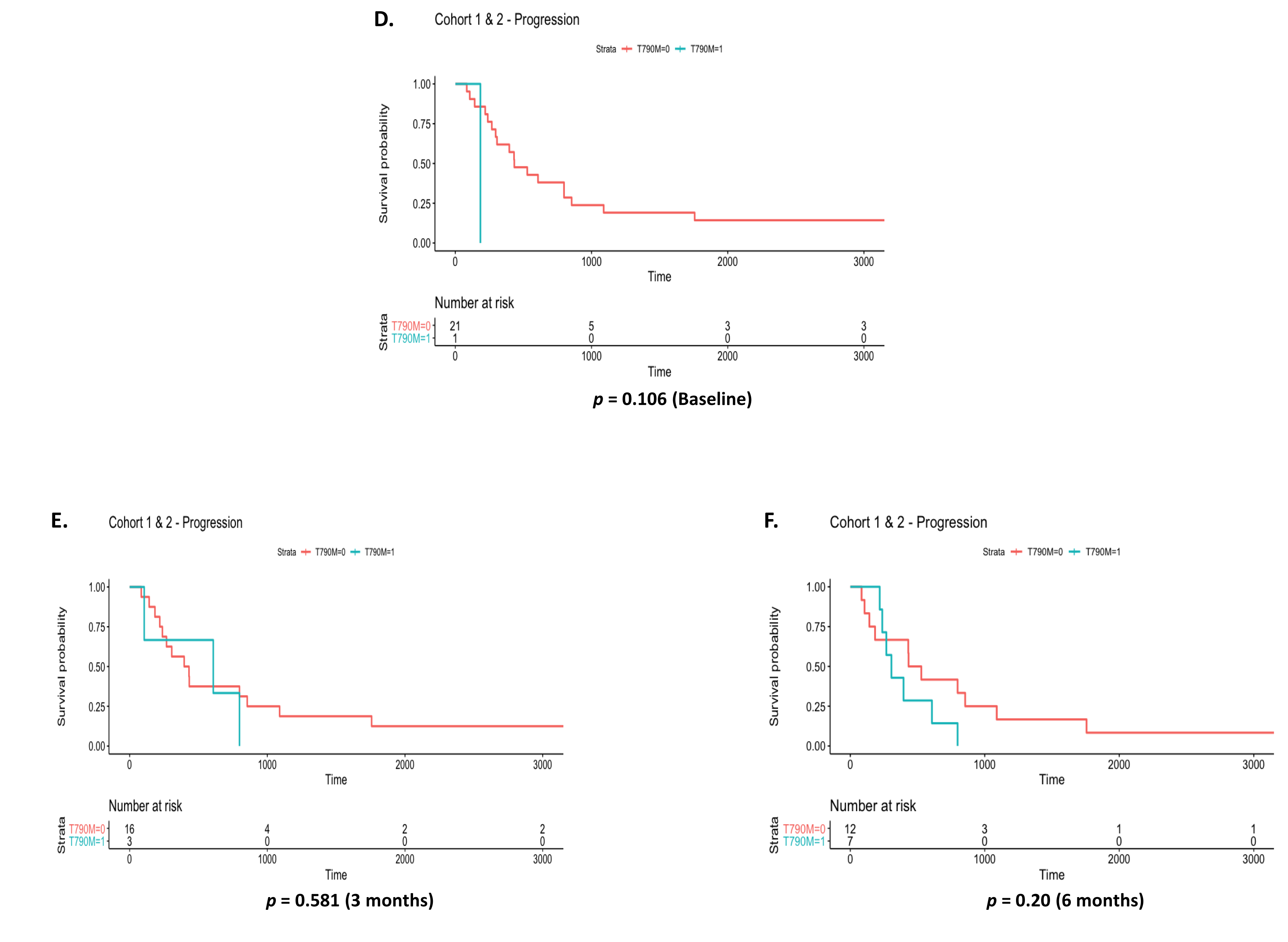

Supplement: Supplementary file 1 [file diagnostics-12-02360-s001.zip › Supplementary Figure S1D_F_Proof_Corrected.tif]

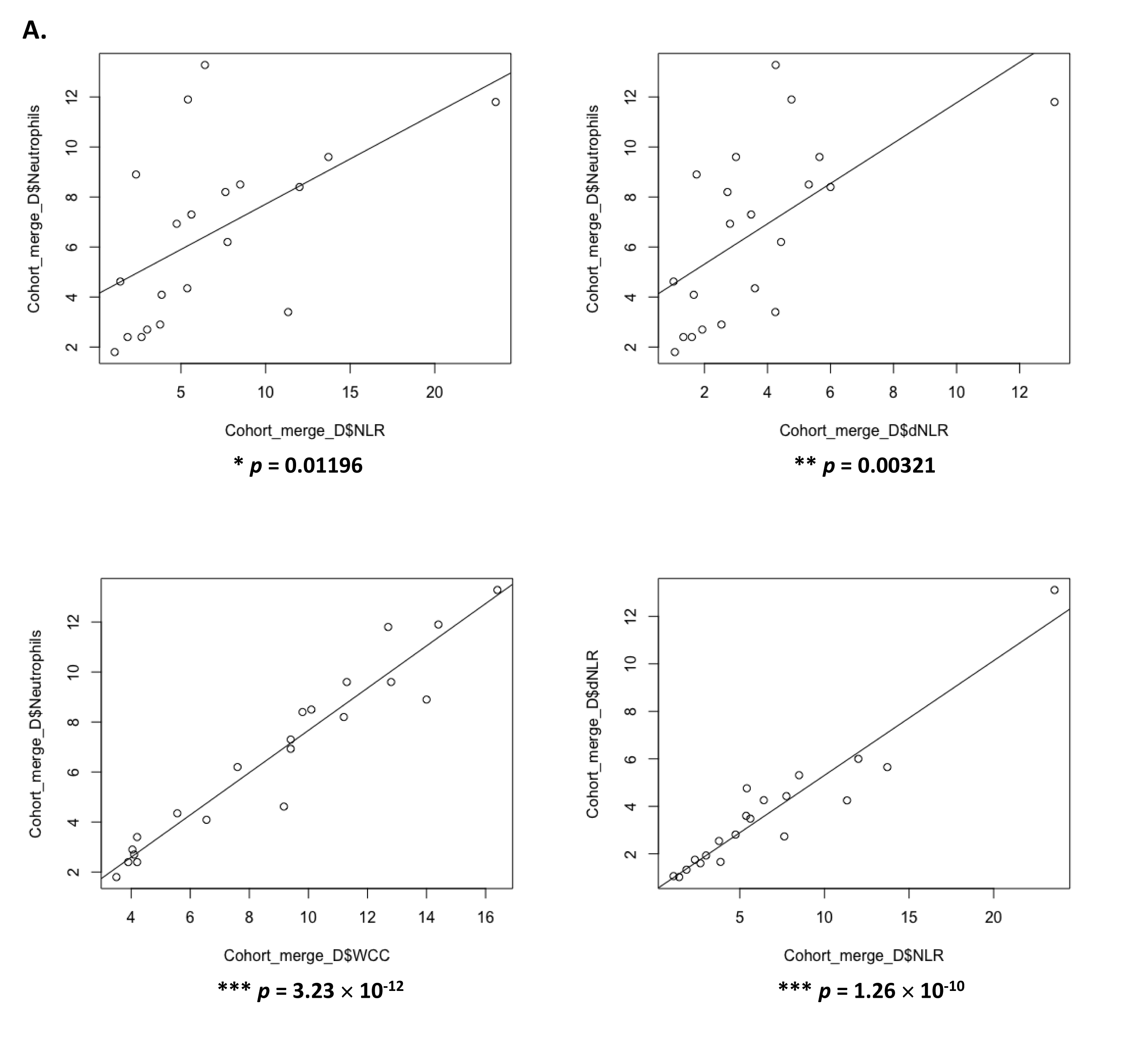

Supplement: Supplementary file 1 [file diagnostics-12-02360-s001.zip › Supplementary Figure S2A_Proof_Corrected.tif]
